# Cell surface nucleolin interacts with and internalizes *Bothrops asper* Lys49 phospholipase A_2_ and mediates its toxic activity

**DOI:** 10.1038/s41598-018-28846-4

**Published:** 2018-07-13

**Authors:** Maria Lina Massimino, Morena Simonato, Barbara Spolaore, Cinzia Franchin, Giorgio Arrigoni, Oriano Marin, Laura Monturiol-Gross, Julián Fernández, Bruno Lomonte, Fiorella Tonello

**Affiliations:** 1 Istituto di Neuroscienze, CNR, Via Ugo Bassi 58/B, 35131 Padova, Italy; 2 Dipartimento di Scienze del Farmaco, Università di Padova, Via F. Marzolo, 5, 35131 Padova, Italy; 3 Dipartimento di Scienze Biomediche, Università di Padova, Via Ugo Bassi 58/B, 35131 Padova, Italy; 4 Centro di Proteomica, Università di Padova e Azienda Ospedaliera di Padova, Via G. Orus 2/B, 35129 Padova, Italy; 5 Instituto Clodomiro Picado, Facultad de Microbiología, Universidad de Costa Rica, 11501 San José, Costa Rica

## Abstract

Phospholipases A_2_ are a major component of snake venoms. Some of them cause severe muscle necrosis through an unknown mechanism. Phospholipid hydrolysis is a possible explanation of their toxic action, but catalytic and toxic properties of PLA_2_s are not directly connected. In addition, viperid venoms contain PLA_2_-like proteins, which are very toxic even if they lack catalytic activity due to a critical mutation in position 49. In this work, the PLA_2_-like *Bothrops asper* myotoxin-II, conjugated with the fluorophore TAMRA, was found to be internalized in mouse myotubes, and in RAW264.7 cells. Through experiments of protein fishing and mass spectrometry analysis, using biotinylated Mt-II as bait, we found fifteen proteins interacting with the toxin and among them nucleolin, a nucleolar protein present also on cell surface. By means of confocal microscopy, Mt-II and nucleolin were shown to colocalise, at 4 °C, on cell membrane where they form Congo-red sensitive assemblies, while at 37 °C, 20 minutes after the intoxication, they colocalise in intracellular spots going from plasmatic membrane to paranuclear and nuclear area. Finally, nucleolin antagonists were found to inhibit the Mt-II internalization and toxic activity and were used to identify the nucleolin regions involved in the interaction with the toxin.

## Introduction

Secreted PLA_2_s (sPLA_2_s) are proteins of about 14 kDa with a conserved tridimensional structure composed of three main alpha helices, a beta sheet and seven disulphide bonds. They have been isolated for the first time from cobra venom and successively from mammalian pancreas, but they are present in about all mammalian tissues. They are major components of snake venoms, and can have different toxic activities depending on their sequence. Among snake PLA_2_s there are hemostasis-impairing toxins, neurotoxins, and myotoxins. They have a high homology with mammalian sPLA_2_s, suggesting that they probably share cellular mechanisms and molecular interactors^1,2^. As an example, the first mammalian sPLA_2_ receptor, PLA2R1, was identified by cross-linking experiments involving OS2, a PLA_2_ from the snake *Oxyuranus scutellatus* that displays both neurotoxic and local myotoxic activities^3^. This is of high relevance, in the light of the emerging involvement of mammalian sPLA_2_s in many human disorders^4–6^.

Most myotoxic PLA_2_s cause a local myonecrosis at the site of snakebite, but some of them act systemically, causing widespread muscle damage. Systemic myotoxins probably have high specificity for a muscle receptor, while locally-acting myotoxins, which induce myonecrosis only locally and at relatively high doses, appear to interact with low-affinity acceptors that retain the toxins at the injection site^7^. Moreover, some local myotoxins also bind to and affect different types of cells, indicating that their acceptors are non-muscle-specific^8^. Notwithstanding the many efforts made by several laboratories to identify myotoxic PLA_2_s receptors/acceptors in cell membranes, this search is still ongoing. In addition, the internalization and possible interaction of these toxins with intracellular targets have not been explored^1^.

A large subfamily of natural variants of snake PLA_2_s have no enzymatic activity, since they have a critical mutation at position 49: the aspartic acid is substituted by another amino acid (lysine in most cases), resulting in the impossibility to coordinate the calcium ion essential for catalysis. Despite the lack of catalytic activity, these PLA_2_ homologues show a high myotoxicity and other toxic effects^1,9^. *Bothrops asper* myotoxin II (Mt-II) is a Lys49 PLA_2_ homologue protein acting as a local myotoxin, but also affecting a wide variety of cell types *in vitro*^8^, including macrophages^10^.

The currently held view is that Mt-II and other PLA_2_-like myotoxins exert their toxic activity by affecting the plasma membrane integrity by interaction with membrane lipids, with consequent rapid influx of calcium ions that eventually triggers a series of degenerative events^11^. In fact, even though Lys49 proteins are catalytically inactive, they retain all the structural characteristics of PLA_2_s, their interfacial binding surface^9^, and therefore the recognition of particular membrane phospholipids, or phospholipid domains, can be a reasonable explication of their toxic activity^1,9^. In particular, most snake myotoxic PLA_2_s have high affinity for anionic phospholipids, as they are basic proteins (the theoretical pI of Mt-II is 9.1), and many of them have been reported to interact with phosphatidylserine^12,13^. We should however consider that some human sPLA_2_s also have a very high isoelectric point, e.g. the theoretical pI of PLA2G2A, the human sPLA_2_ with higher homology to Mt-II^1^, is of 9.38, so myotoxic PLA_2_ are expected to possess some other characteristic, besides net charge, that makes them so toxic.

The interaction with membrane lipids hardly explains the specificity of some myotoxins for muscle cells, and for myotubes in comparison to myoblasts, and the Mt-II triggering of specific intracellular signaling pathways. In fact, immediately after addition to cell cultures, Mt-II induces calcium release from intracellular stores, followed by the opening of potassium and ATP channels, activation of purinergic receptors, and finally massive entry of calcium from extracellular medium^10,14,15^. Moreover, many inhibitors of these signal pathways, e.g. purinergic inhibitors, inositol triphosphate signalling inhibitors and intracellular calcium chelators, block or slow down the action of toxin^10^. A possible explanation is that Mt-II, interacting with a membrane protein receptor, triggers a signalling that leads to the modification of the membrane lipid composition with consequent destabilizing action of the plasma membrane by the toxin, and massive calcium entry. Alternatively, the triggered signal could lead to toxin internalization, and the Mt-II cell entry could be a necessary step for its toxic activity.

To help clarify the action of snake myotoxins on target cells, in this work we conjugated Mt-II, purified from *Bothrops asper* venom, with a fluorophore to investigate its cellular localization, and with biotin to use it as bait to isolate its protein interactors. By fluorescence microscopy, the toxin was found to be internalized in mouse myotubes and in RAW264.7 macrophages, and transported to their perinuclear and nuclear zone. By protein pull-down and mass spectrometry, Mt-II was found to interact with nucleolin (NCL), a multifunctional protein with a high percentage of disordered domains^16^. NCL is a nucleolar protein but, in response to particular stimuli or during the different phases of the cell cycle, it can also localize in nucleoplasma, cytoplasm and on the cell surface^17^. Furthermore, cell surface NCL was reported to interact with and mediate the internalization of different types of molecules^17,18^.

The interaction between Mt-II and NCL was confirmed with confocal microscopy. The two proteins were found to colocalise in, Congo red sensitive, cell surface molecular assembly at 4 °C, a temperature in which the endocytosis is inhibited, and in cytosolic, paranuclear and nuclear area structures at 37 °C.

The involvement of NCL in Mt-II internalization and toxic activity was verified, in RAW264.7 and mouse primary macrophages, with experiments of Mt-II cellular uptake, and cytotoxicity test in presence of an anti-NCL rabbit polyclonal antibody, and of AS1411, an aptamer that binds specifically to NCL^19^. In addition, we observed that, by lowering NCL expression by RNA interference in Hela cells, the sensitivity of these cells to Mt-II cytotoxicity is considerably decreased. Finally, thanks to competitors that bind to different regions of NCL, we identified central RRM and the C-terminal R/F-GG domain of NCL as the regions involved in the interaction with Mt-II.

## Results

### Mt-II conjugation by transglutaminase with a fluorescent or biotinylated glutamine-donor peptide

The reaction of Mt-II with transglutaminase^20^ (TGase) in presence of a TAMRA conjugated or a biotinylated glutamine-donor peptide allowed us to obtain mono-derivative Mt-II with conserved toxic activity. We purified the monoderivatives by RP-HPLC (supplementary Fig. S1) and characterized them by ESI-mass spectrometry (Table 1 and supplementary Fig. S2). The activity of the modified toxin was verified with a cytotoxicity test on RAW264.7 cells and resulted to be almost completely conserved (Table 1). The slight loss of activity may be due to the purification and freeze-drying steps leading to oxidation of the protein (see Figure S2).

**Table 1:** Measured molecular masses and relative cytotoxic activity of the Mt-II derivatives produced upon incubation with TGase in the presence of Z-Gln-Gly-CAD-Biotin or Z-Gln-Gly-CAD-TAMRA.

Protein species	Modification	Molecular mass (Da) ± SD		Relative cytotoxic
	(number)	Found^a^	Calculated^b^	activity ± SD^d^
Mt-II		13725.48 ± 0.08	13725.02	100 ± 6%
		13759.38 ± 0.14	13759.04^c^	
Mt-II	Biotin (1)	14355.18 ± 0.05	14355.79	84 ± 7%
		14388.98 ± 0.11	14389.81^c^	
Mt-II	TAMRA (1)	14542.67 ± 0.45	14541.93	81 ± 5%
		14576.91 ± 0.06	14575.95^c^	

^a^Experimental molecular masses determined by ESI-MS.

^b^Calculated average molecular masses.

^c^Isoform L114F of Mt-II^60^.

^d^Percentage of cell death obtained with the modified Mt-II, with respect to that obtained with the wild type protein. RAW264.7 cells plated in p6, 5·104 cells/well, were treated with Mt-II, Mt-II-TAMRA, or Mt-II-Biotin (20 µg/ml). After one hour of incubation at 37 °C, 5% CO2, cell vitality was measured with Cell Titer 96 tetrazolium assay (Promega). The results are the mean of three independent experiments.

### Mt-II-TAMRA is internalised in mouse myotubes and macrophages

**Figure 1:**
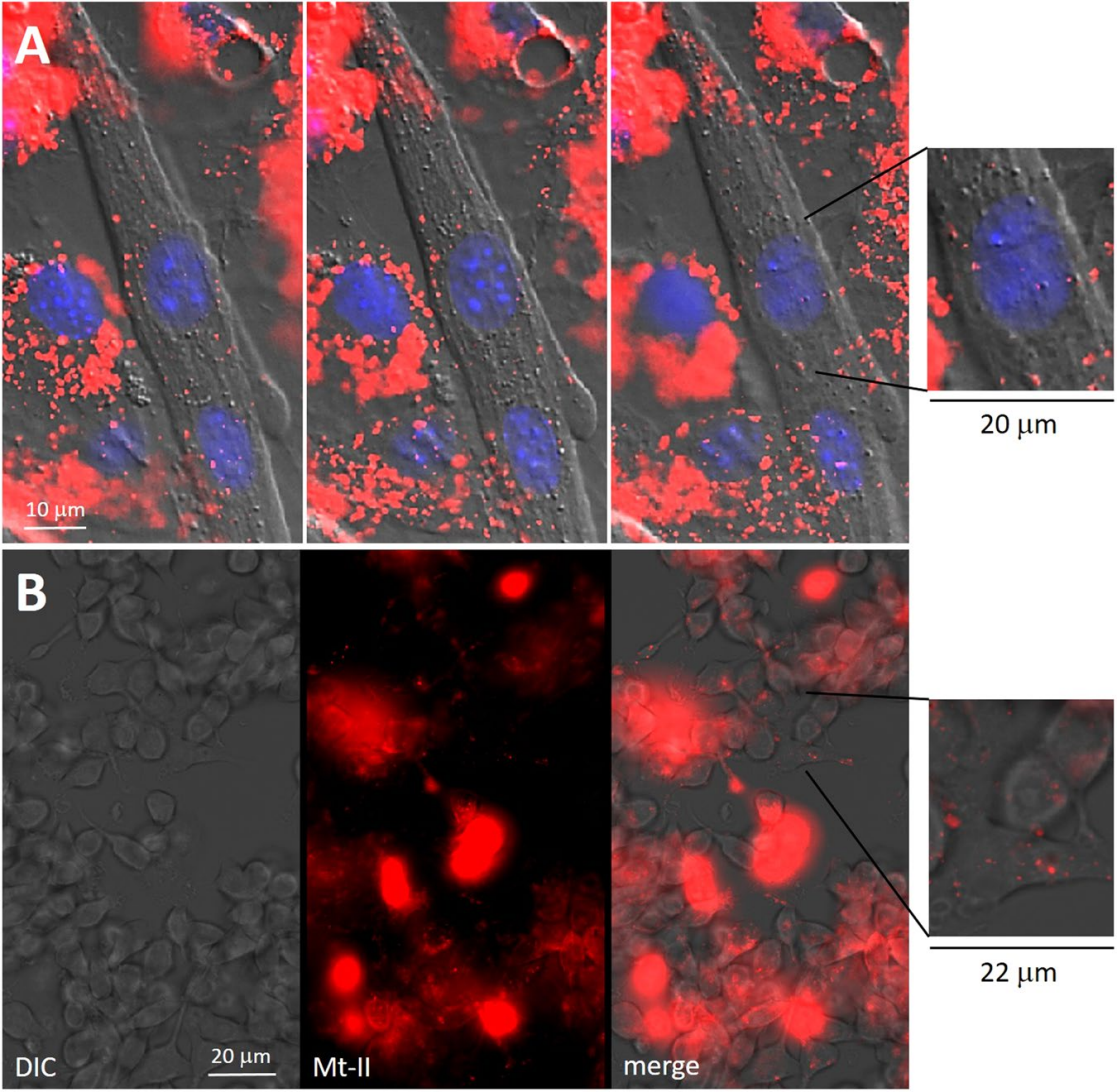
Internalization of Mt-II in myotubes and macrophages. (**A**) Mouse primary myotubes were treated with Hoechst 33342 (1:1000) to stain the nuclei, intoxicated for 30′ with Mt-II-TAMRA (15 µg/ml), washed and observed. Three observation planes were reported, having a distance of 1.5 µm between them, to highlight that Mt-II is present inside the cells. (**B**) RAW264.7 cells were treated for 30′ with Mt-II-TAMRA (15 µg/ml), washed and observed. The observed fields are representative of what happens in the intoxicated cultures: single cells are intoxicated in different times, so some cells appear morphologically altered and full of the toxin, while in other cells the toxin is present in few points. The inserts show a detail of cells morphologically unaltered but containing some toxin spots. For the intoxication kinetic, see Supplementary Fig. S3. Images were acquired with wide-field epifluorescence and transmitted light microscope (DIC Nomarski).

Mt-II-TAMRA added to culture of primary mouse myotubes and of RAW264.7 macrophages is internalized in both kinds of cell (Fig. 1). Thirty minutes after the addition of Mt-II-TAMRA, in the same observation field, some cells appear full of the toxin, while others show few toxin spots close to the plasma membrane and in perinuclear and nuclear area. Mouse primary myotubes containing a higher quantity of internalized toxin show prominent nuclei and strongly damaged plasma membrane, while, the cells with few internalized toxin appear to be morphologically unaltered (Fig. 1A). Toxin full RAW264.7 cells, at contrast phase DIC images, appear rounded and with disintegrated plasma membrane, while cells containing less toxin have a normal morphological aspect (Fig. 1B). This dishomogeneous aspect of the cell cultures intoxicated with Mt-II is due to the kinetic detail of the Mt-II intoxication process. As reported in Tonello *et al*.^10^ and as is possible to observe in the supplementary Fig. S3A, after adding of the toxin some cells respond before others with an apparently random trend. The process of intoxication of a single cell, i.e. the time from when a small amount of toxin enters the cell to when the cell shows obvious morphological changes and a higher amount of internalized toxin, is of few minutes (3–10 minutes, Figure S3). The death of the entire culture takes from one to several hours, depending on the amount of toxin used^8,10^.

### Isolation and identification of Mt-II protein interactors from macrophage cell extracts

**Figure 2:**
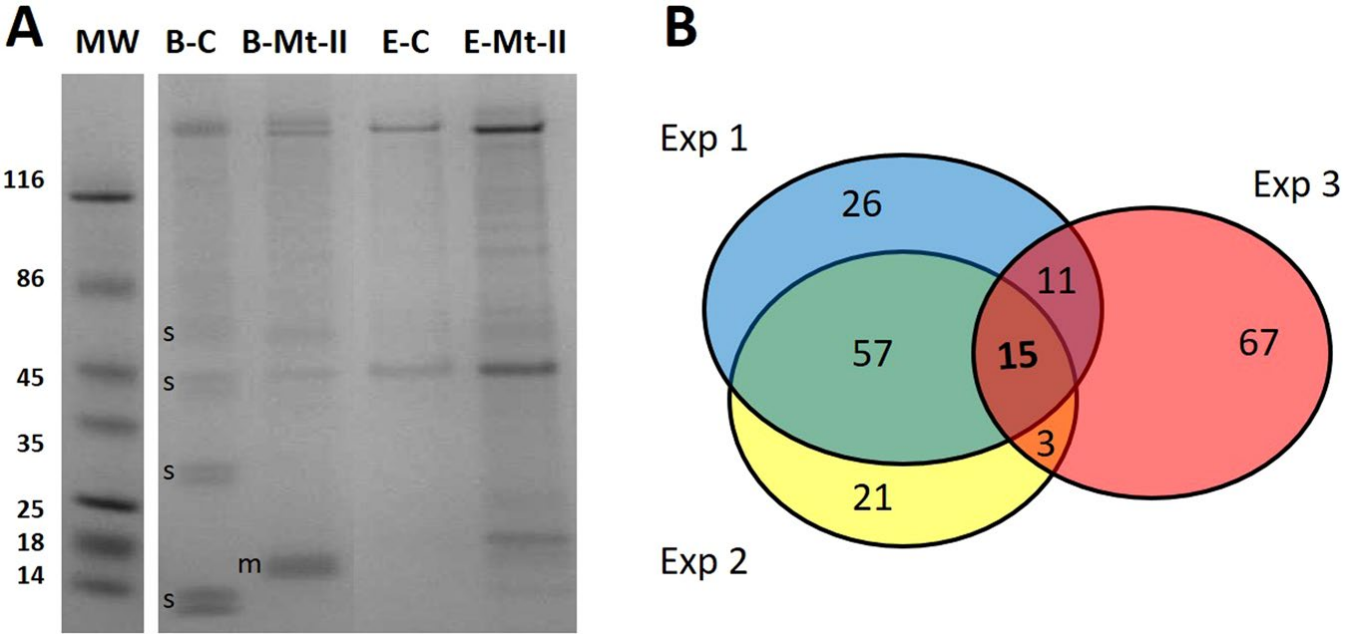
Pull down and mass spectrometry analysis of Mt-II protein interactors. (**A**) SDS-PAGE analysis of pull down experiment with biotinylated Mt-II (Mt-II-B) combined with streptavidin magnetic beads as bait and an extract of RAW264.7 as prey. As control the pull down was executed with the resin without Mt-II-B. B-C and B-Mt-II: control and Mt-II bound beads after elution of fished proteins; E-C and E-Mt-II: samples eluted from control and Mt-II combined streptavidin magnetic beads. The bands indicated with ‘s’ are due to streptavidin detached from the magnetic beads, the band indicated with ‘m’ correspond to the biotinylated Mt-II. Other bands present in control sample lanes are due to proteins that bind a-specifically to the streptavidin magnetic beads and to endogenous biotinylated proteins. Eluted samples were subjected to in-gel digestion followed by LC–MS/MS and semi-quantitative data analysis (Tables S1–S3). The experiment was repeated two times with the same conditions, and a third with the addition of a crosslinking step to isolate proteins closer to the bait. (**B**) Venn diagram of the number of proteins identified in the three pull down experiments. Among the fifteen proteins present in all three experiments (Table S4) NCL was identified with at least 13 unique peptides and an intensity ratio, sample over control, higher than 45.

Biotinylated Mt-II (Mt-II-B) was combined to streptavidin magnetic beads and utilized as bait to isolate interacting proteins from a suspension of mechanical lysed RAW264.7 cell extract. The use of magnetic particles avoids the need to solubilize the sample in detergents, and/or other stages of sample pretreatment, and this makes the purification more gentle and effective, since the molecules are kept in a more natural state^21^. After the binding an extensive washing was applied to remove aspecifically bound proteins. The isolated proteins were eluted with a solution that does not detach the biotinylated myotoxin from the resin (5% SDC), then digested with trypsin and identified by LC-MS/MS analysis. As controls, the same procedure was performed using beads without Mt-II. The experiment was repeated twice under the same conditions, each time leading to the identification of about one hundred proteins, each of them with a false discovery rate (FDR) less or equal to 0.01, and with at least four sequenced unique peptides (Tables S1 and S2). To identify proteins closer to the bait, the experiment was repeated a third time by adding a crosslinking step on streptavidin beads, after protein isolation. The crosslinker (DTSSP) used contains amine-reactive NHS-ester ends around a 12 Å, 8-atom spacer arm, and a central disulfide bond that can be cleaved with reducing agents. After the crosslinking reaction, the non-covalently bound proteins were removed with extensive washing and the crosslinked proteins were detached by reduction with DTT. This procedure allowed us to identify again one hundred proteins, but with a lower abundance: we identified only 21 proteins with a sample/control intensity ratio higher than 3 (Table S3), in comparison to 111 and 84 of the first and second experiment. Fifteen proteins were found in common in all three experiments (Fig. 2 and Table S4), including NCL. The analysis of the list of the fifteen identifiers in Human Mine^22^ revealed several interesting aspects, including: physical interactions among these proteins has been already reported; extracellular matrix, ribonucleoprotein complex, focal adhesions and cell-substrate adherent junction are the more significant GO terms in cellular component gene ontology enrichment analysis; all fifteen proteins have been found in a proteomic analysis of α4β1 integrin adhesion complexes.

NCL was identified in all fishing experiments among the proteins with the largest number of unique peptides and with an estimated abundance at least 45 times higher than in the control sample. As NCL is a nucleolar protein known to be present also on the cell surface, and to mediate the internalization of many extracellular factors, we decided to deepen the study of the interaction between Mt-II and this protein.

### Mt-II pulls down NCL from RAW264.7, C2C12 and *exvivo* muscle membrane preparations

NCL is one of the most abundant non-ribosomal proteins of the cell. It is mostly localized in nucleolus (90%) and nucleus (about 5%), but it is present also in cytosol and cell membrane in variable concentrations, depending on cell status and on NCL post translational modification^17^. To confirm the interaction between Mt-II and NCL in cell types other than macrophages, and to clarify if Mt-II interacts with NCL present in cell membrane, we prepared an extract of membranes from RAW264.7, C2C12 myotubes and from *ex-vivo* muscle. Mt-II-biotin, bound to streptavidin magnetic beads, was found to pull down NCL from membrane extracts obtained from all three preparations (supplementary Fig. S4).

### Mt-II co-localizes with NCL in cell surface assemblies sensitive to Congo red staining

**Figure 3:**
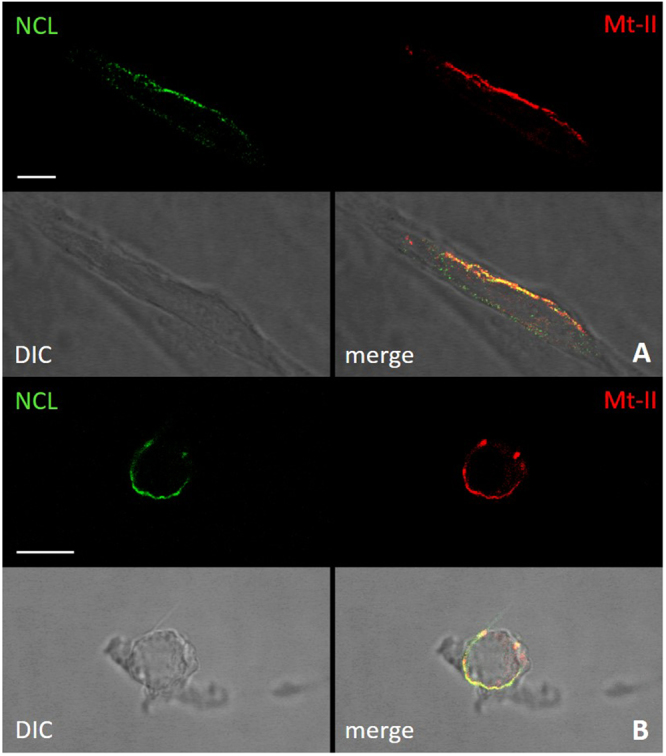
Colocalization of NCL and Mt-II in cell membrane of myotubes and macrophages. Mouse primary myotubes (**A**) and RAW264.7 cells (**B**) were incubated with anti-NCL (rabbit) (45′, RT), then treated with Mt-II-TAMRA (15 µg/ml, 15′, 4 °C). After extensive washings in PBS, cells were fixed, treated with an anti-rabbit Ig secondary antibody Alexa Fluor 488-conjugated and visualized by confocal microscopy. Scale bars correspond to 10 µm.

**Figure 4:**
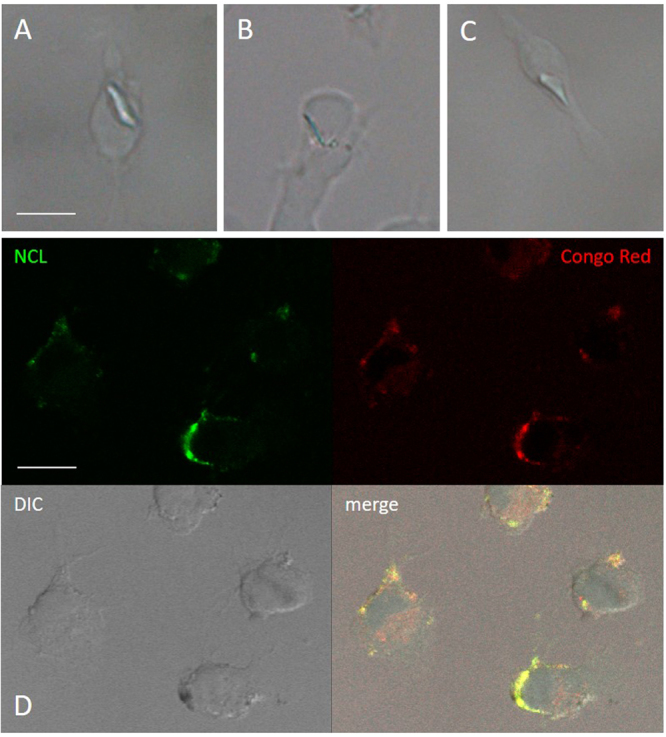
Congo red staining of Mt-II assemblies in cell membrane. RAW264.7 cells were treated with Mt-II (14 µg/ml, 20′, 4 °C), colored with Congo red (10′, 4 °C) fixed and observed in polarized light (**A–C**) or treated with rabbit polyclonal anti-NCL (45′, RT), with Mt-II (14 µg/ml, 20′, 4 °C), colored with Congo red, fixed, treated with an anti-rabbit secondary antibody Alexa Fluor 488-conjugated and observed with a confocal microscope (**D**). Scale bars correspond to 10 µm. Mt-II forms cell surface assemblies that, after Congo red staining, show a green birefringence in polarized light and a red color at visible light (λ_exc_ = 543 nm) that colocalize with the staining of NCL.

The interaction between NCL and Mt-II in cell surface was verified also by co-localization experiments. In developing the experimental protocol, we observed that the amount of NCL present in the cell surface of non-stimulated cells is very low (Fig. S6), but it increases in live cells treated with the toxin or with an anti-NCL antibody, at RT or 37 °C. In fact NCL is released to the surface through an unconventional secretion that does not go through the classic ER-Golgi pathway^23^. Since we aimed at observing the interaction between NCL and Mt-II on the cell surface, we decided to add the toxin at low temperature (4 °C) to prevent its internalization and consequent cell death. Thus, we treated cells with the anti-NCL antibody at RT to stimulate the secretion of NCL and successively with Mt-II-TAMRA at 4 °C. Finally, we fixed the cells, treated them with the secondary antibody and we acquired images by confocal microscopy. The images obtained (Fig. 3) show that NCL and Mt-II colocalize in long stretches, quite thick, on the cell surface. This kind of staining, non-dotted but with bigger areas, is typical of proteins involved in phase transition phenomena that give rise to membrane-less organelles^16,24^. Membrane-less organelles are molecular assemblies that form through multivalent weak interactions and that mediate diverse biological processes. The nucleolus is an example of this kind of organelles and NCL is one of its main components^25^. Phase transition phenomena can happen also on the cell membrane, for example to trigger signaling events^26^. As sPLA_2_s were reported to form amyloid-like fibrils when they come in contact with phospholipid bilayers^27^, and prion-like interactions are involved in phase transition phenomena, we decided to assess if Mt-II assemblies on cell membrane are sensitive to Congo red, an amyloid specific dye. On the surface of cells intoxicated with Mt-II and treated with Congo red, green birefringence on polarized light, and red colored zone that co-localize with NCL  were observed, both signs of the presence of amyloid-like fibrils (Fig. 4). Assemblies formed by Mt-II are not SDS-resistant: SDS-PAGE analysis of extracts from cells incubated with Mt-II-B and a membrane impermeable crosslinking agent shows that Mt-II forms high molecular weight complexes only in crosslinked samples (supplementary Fig. S7). Since proteins that form amyloid-like fibrils are characterized by the presence of prion-like domains^16,24^, we inspected the primary structure of Mt-II for the presence of prion-promoting amino acids. We found four traits rich in tyrosine, serine and glycine with some asparagine or glutamine, amino acids enriched in human prion-like domains, with in addition the presence of proline and charged amino acids that are relevant to modulate the formation of prion aggregates^28^. Interestingly, these traits are located in exposed loops of Mt-II (supplementary Fig. S8) and homologous proteins, sites that can undergo conformational changes in an otherwise very stable tertiary structure.

### Mt-II colocalises with intracellular NCL

**Figure 5:**
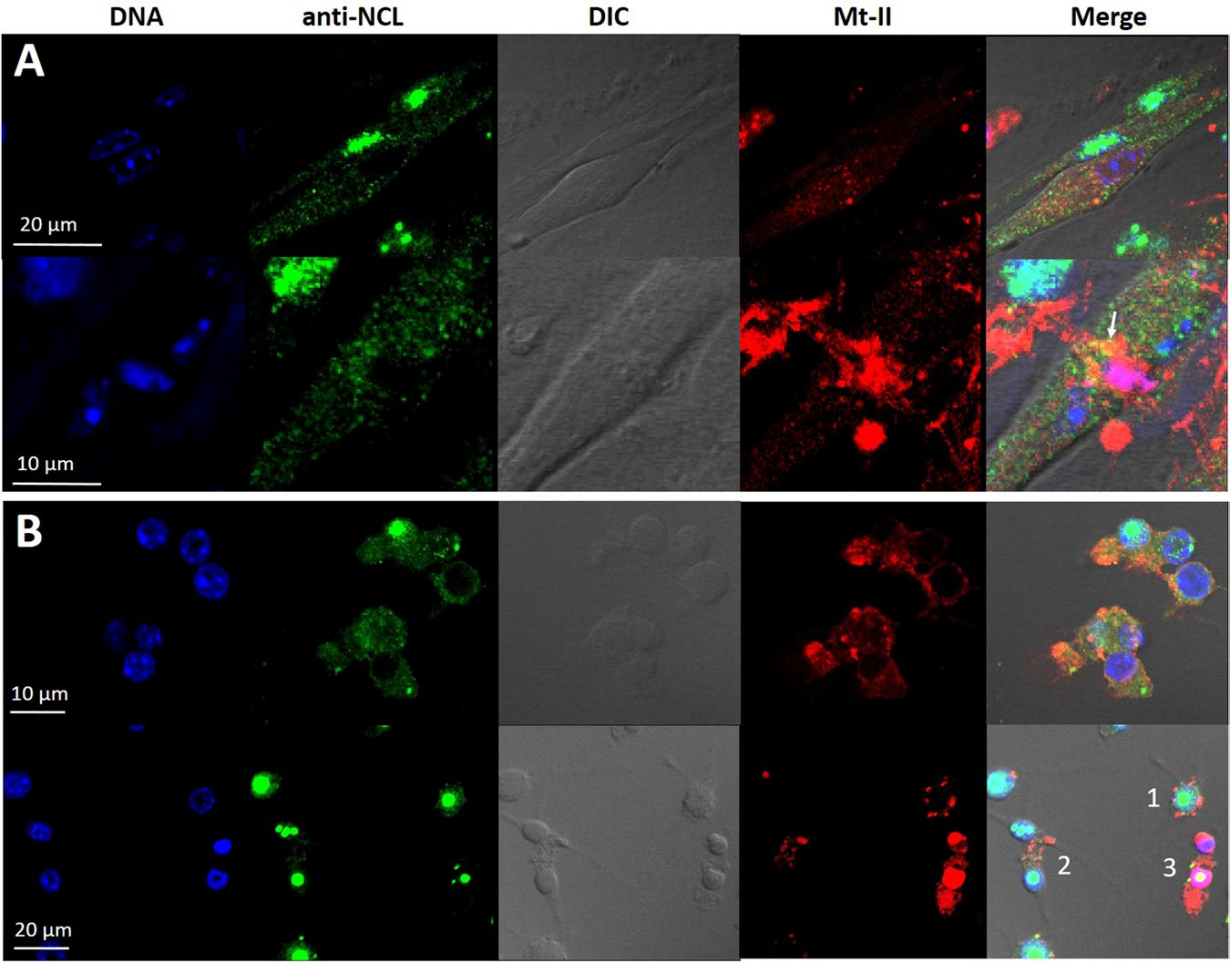
Intracellular colocalization of NCL and Mt-II in myotubes and macrophages. Mouse primary myotubes (**A**) and RAW264.7 cells (**B**) were intoxicated with Mt-II-TAMRA (15 µg/ml) for 20′, fixed, permeabilized with 0.5% Triton in PBS, and treated with a rabbit polyclonal anti-NCL and with an anti-rabbit Ig secondary antibody Alexa Fluor 488-conjugated and visualized by confocal microscopy. Nuclei were stained with the nuclear fluoro-probe Hoechst 33342. The arrow in panel A indicates points where NCL seems to surround the toxin. Numbers in panel B indicate different stages of cell intoxication: the toxin induces the formation of superficial blebs where is present also NCL (1), is internalized in structures containing also NCL (2), and finally massively penetrates the cell, occupying the entire nucleus (3).

Mt-II-TAMRA internalized in mouse primary myotubes colocalises with NCL (Fig. 5A and supplementary Fig. S9A) in spots around the nucleus. In some cells the toxin is found inside the nucleus and colocalizes with DNA (violet area). In some points the toxin seems to be present in structures surrounded by NCL (see arrow in Fig. 5A). Mt-II-TAMRA colocalizes with NCL also in RAW264.7 cells (Fig. 5B and supplementary Fig. S9B). In Fig. 5B cells at different stages of intoxication are observed: the toxin induces the formation of superficial blebs where NCL is also present (1), is internalized in structures containing NCL as well (2), and finally massively penetrates the cell, occupying the entire nucleus (3). In control images (supplementary Fig. S10) NCL, as expected, is concentrated mainly in the nuclei, around DNA, while little of it is dispersed in the cytosol and plasma membrane. Interestingly, in both types of cells, the treatment with toxin increases the amount of NCL diffused in the cytosol and present in the cell membrane, as can be seen by comparing Fig. 5A,B with their controls (supplementary Fig. S10).

### Anti-NCL antibody, a NCL specific aptamer and NCL RNAi inhibit Mt-II toxic action

**Figure 6:**
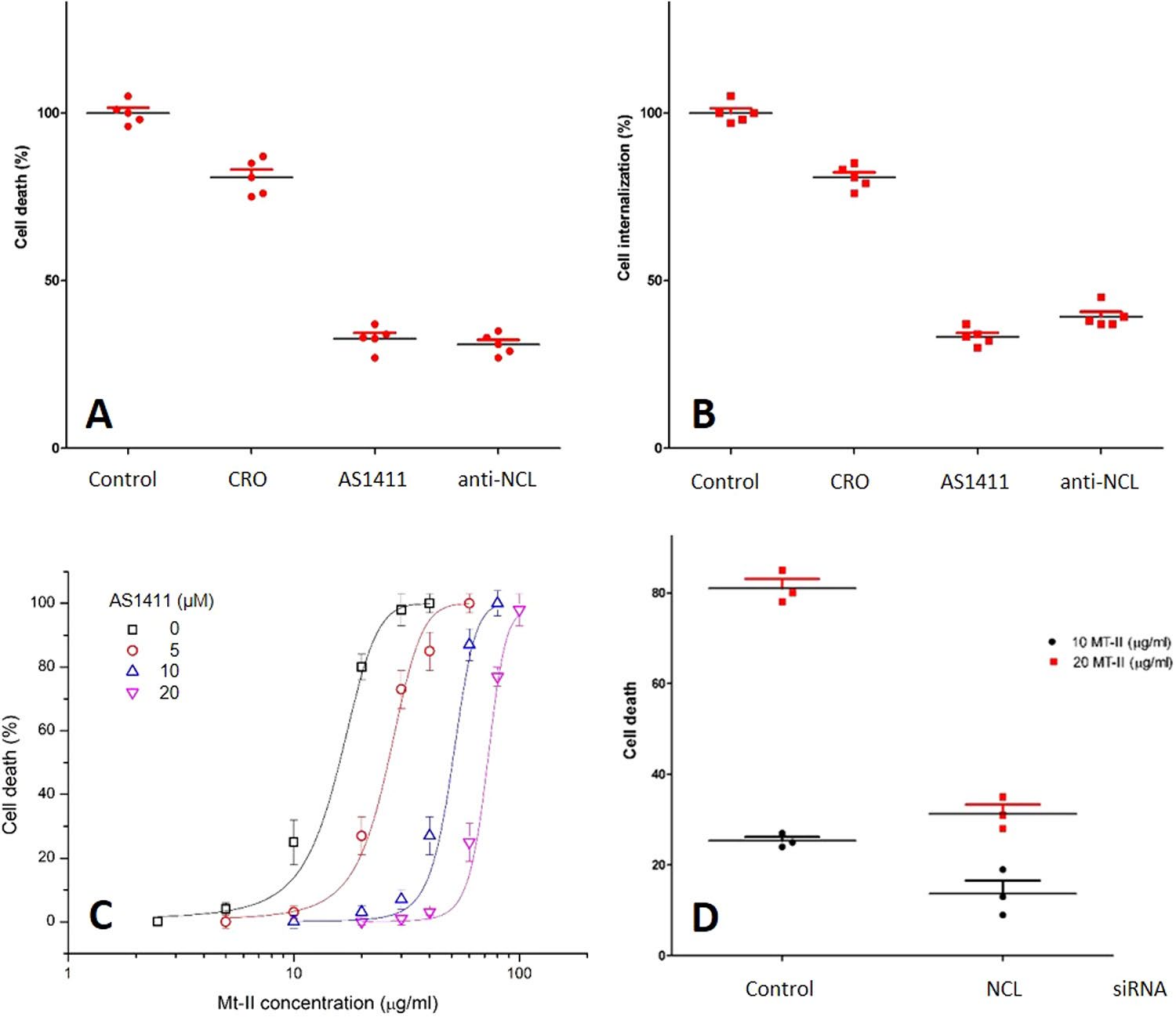
Inhibitory activity of NCL antagonists in cells intoxicated with Mt-II. Mouse primary myotube cells were treated with Mt-II (20 µg/ml) for 30′ at 37 °C or with Mt-II-TAMRA (15 µg/ml) for 10′, 37 °C and checked for their vitality (**A**) or for the internalization (**B**) of the fluorescent toxin. The experiments were repeated in presence of the NCL specific aptamer AS1411 (5 µM), a control aptamer (CRO, 5 µM) and an anti-NCL antibody (20 µg/ml), and the percentage of residual activity was calculated respect to control (100%). (**C**) Dose dependence curves of Mt-II cytotoxicity in mouse primary myotubes in presence of different quantities of the aptamer AS1411. Reported points are the mean of three independent experiments. (**D**) Cell death of Hela cells treated with siRNA trilencer specific for human NCL and a non-targeting duplex siRNA control, and intoxicated with two different concentration of Mt-II. In the range graphs each point represents the result of a single experiment, the larger bars represent the mean. Error bars represent SD.

To understand if the interaction between NCL and Mt-II has a role in Mt-II internalization and toxic activity, we measured the quantity of toxin internalized and its cytotoxicity in the presence of AS1411, an aptamer that binds specifically NCL^19^, a control aptamer (CRO), and an anti-NCL antibody. We performed an internalization assay intoxicating target cells with Mt-II-TAMRA, washing extensively to remove unbound protein, and measuring the fluorescence after cell lysis. For the cytotoxicity assay, cells were incubated with unlabeled Mt-II and the percentage of cell death was measured with a colorimetric assay for assessing cell metabolic activity, in the case or RAW264.7 cells, or by measuring the release of LDH, a plasma membrane damage index, in the case of the myotubes. The results of the two assays are reported in Fig. 6A,B and supplementary Fig. S11: the two NCL competitors substantially inhibited both the internalization and the toxicity of Mt-II. The inhibition of the AS1411 aptamer was further studied by performing vitality experiments on primary myotubes in presence of different concentrations of aptamer and Mt-II (Fig. 6C), observing that at higher doses AS1411 inhibits completely the cell death induced by the toxin.

Given the broad cell cytotoxicity of Mt-II^8^, we chose Hela cells as model to text the effect of NCL silencing on Mt-II toxicity, because the transfection in these cells is more efficient and less harmful than in myotubes and macrophages. NCL expression in Hela was reduced, with specific siRNA, to about fifty percent (see Fig. S12), not further because NCL is necessary for cell vitality^17,29^. Subsequently, the cells were treated with two different quantities of Mt-II for 30 minutes. Hela cells with a lower expression of NCL resulted to be more resistant to the treatment with the toxin (Fig. 6D).

### NCL regions involved in interactions with Mt-II

**Figure 7:**
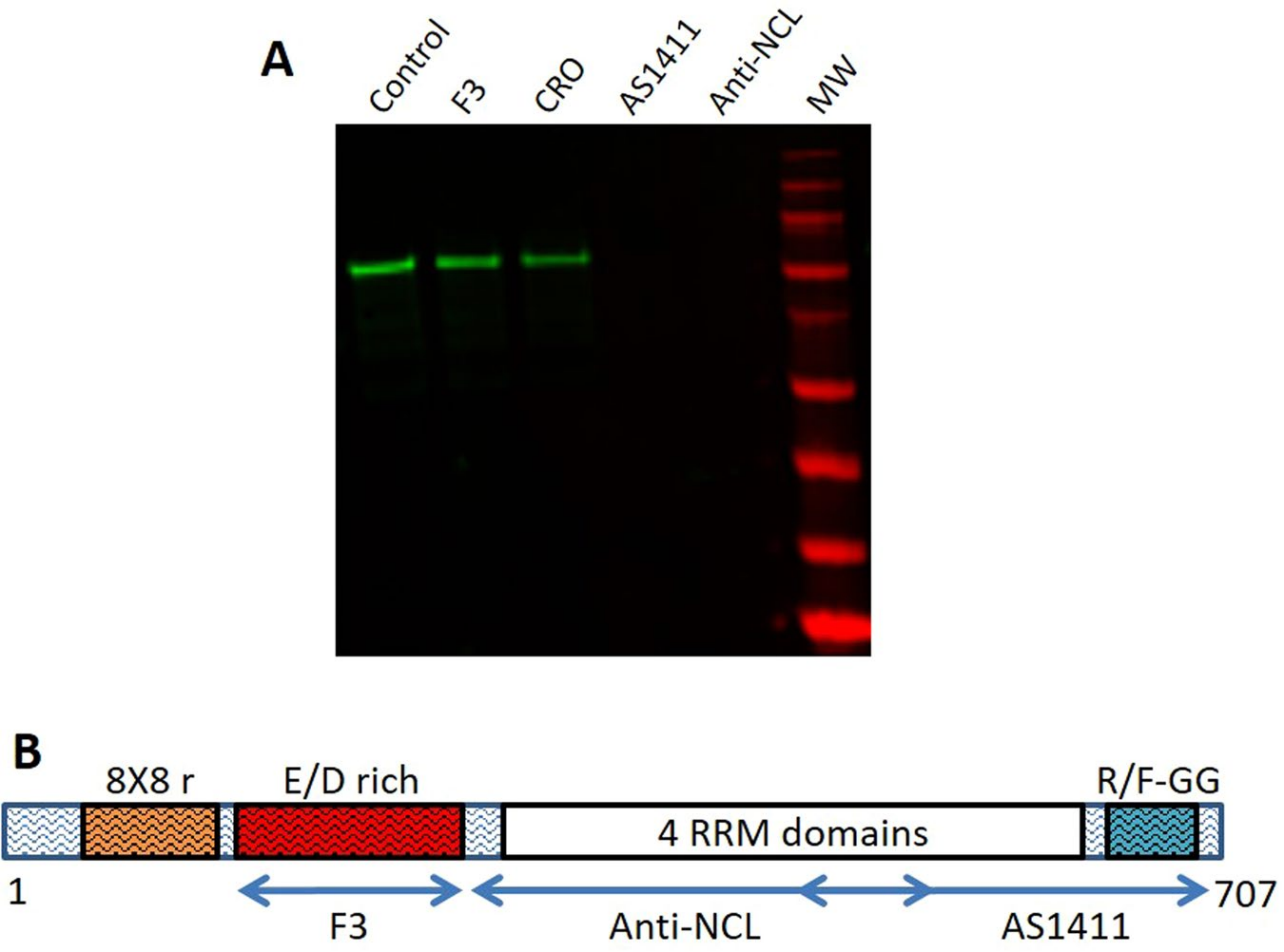
Mt-II interacts with NCL central and C-terminal regions. (**A**) Western blot analysis, marked with anti-NCL, of proteins pulled down by Mt-II-B/streptavidin magnetic beads from RAW264.7 membrane protein extracts alone (control) or in presence of NCL binding molecules: a peptide (F3, 5 µM), an aptamer (AS1411, 5 µM), and an antibody (anti-NCL, 20 µg/ml). CRO is an aptamer used as control (5 µM). (**B**) Representation of the primary structure of mouse NCL and its domains. Regions recognized by the different antagonists used in this work were highlighted with double-headed arrows.

NCL is a protein of 710 (human) or 707 (mouse) amino acids, respectively, composed of three domains: an N-terminal disordered domain rich in negatively charged amino acids, a central domain containing four RNA Recognition Motifs (RRMs), and a C-terminal disordered domain containing R/F-GG repeats (Fig. 7B). Many substances were found to interact with NCL located on the cell surface, in particular on surface of cancer cells where NCL is present at higher concentration, and to trigger an internalization process^17,18^. AS1411 is an anticancer aptamer that binds to the central and C-terminal regions of NCL^19^, and F3 is a tumor-homing peptide that binds to the N-terminal negatively charged domain of NCL^30^. We tested the ability of AS1411, F3 and of an anti-NCL central domain polyclonal antibody to compete for the fishing of NCL by Mt-II-B. The antibody and the aptamer were found to inhibit the fishing of NCL, while F3 did not compete (Fig. 7A and supplementary Fig. S13), indicating that the central and C-terminal regions of NCL are that involved in the interaction with Mt-II.

## Discussion

In this work, by exploiting an enzymatic modification of *B. asper* Mt-II with TGase, we obtained the toxin in fluorescent and biotinylated forms useful for the study of its cellular activity.

The red fluorescent conjugated Mt-II-TAMRA was found to be internalized in myotubes and macrophages and to localize in perinuclear and nuclear area (Fig. 1). This is, to our knowledge, the first study reporting that a snake myotoxic PLA_2_-like protein is internalized in cells. Other snake PLA_2_s have been shown to be internalized, but only the neurotoxic PLA_2_s, in neuronal cells. Notably, notexin, beta-bungarotoxin and taipoxin have been shown to localize in mitochondria of spinal cord motor neurons and cerebellar granule neurons^31^, while ammodytoxin was found into the cytosol, synaptic vesicles, and mitochondria of motoneurons^32,33^. Snake cytotoxins, non-enzymatic three-fingered fold proteins, were found to be internalized in cells, and to induce cell death mainly by interaction with lysosomes^34^. Importantly, human PLA2G2A was also reported to be internalized in monocytes and finally transported into the nucleus^35^, and some secreted mammalian phospholipases, including PLA2G2A, were reported to be present in perinuclear area of several cell types^36^.

The biotinylated Mt-II allowed the isolation of putative RAW264.7 cellular proteins interacting with the toxins. Other snake PLA_2_s were used as bait to isolate protein interactors^2,3^ but not *B. asper* myotoxins. In addition, the use of streptavidin magnetic beads and LC-MS/MS analysis are important innovations in this kind of experiment. The first allowed us to isolate the interacting proteins without the need to solubilize the cell extract in detergent containing solutions, and avoiding centrifugal steps in the incubation and washing phases, making the process more delicate^21^. The second allowed us to identify the isolated proteins all together without having to separate them first. The fishing experiment was repeated three times to distinguish relevant from random interactions, and, the third time, by adding a crosslinking step to enrich proteins physically closer to the bait. We thus identified fifteen proteins as possible Mt-II interactors, that interestingly were all previously found in the proteomic analysis of the adhesion complex of integrin α4β1^37^, a transmembrane protein that binds PLA2G2A^38^.

Among the identified Mt-II protein interactors, given the intracellular localization of the toxin in para-nuclear and nuclear area, we focused our attention on NCL, because it was reported to mediate the internalization of several factors from the cell surface to the nucleus. An anti-NCL antibody allowed us to verify that the two proteins colocalise on both cell surface and inside. We then utilised the same antibody, and an antitumoral aptamer that binds specifically NCL, to demonstrate that the Mt-II/NCL interaction is functional to both Mt-II internalization and cytotoxicity, and that the NCL parts involved in interaction with the toxin are the central RNA-binding and C-terminal regions.

The Mt-II/NCL interaction is remarkable for several reasons. First, NCL is the first Mt-II interacting protein connected to the toxic mechanism. An interaction of Mt-II with the KDR/VEGF receptor 2 has been previously reported^39,40^ but no evidence for a functional role of this interaction was found by using a blocking monoclonal antibody to the receptor or the inhibitor tyrphostin^9^. Secondly, the interaction of Mt-II with NCL may explain pharmacological properties of snake myotoxins. The preferential activity of these toxins against cancer cells^41–44^ can be correlated to the fact that NCL is more abundant on the surface of these cells^17,45,46^. Moreover, some snake PLA_2_s have been reported to have antiviral activity^47,48^ and accordingly NCL is involved in internalization of many viruses^17,18^. Thirdly, NCL is a nucleolar protein the concentration of which, in cell surface, increases only in particular circumstances or after determined stimuli^17,23^ and this fits with the fact that the toxic mechanism of Mt-II is particularly complex and requires different signalling events (see introduction). The NCL secretion mechanism is still unknown, and it is an unconventional mechanism, that does not involve the classical secretion pathway via endoplasmic reticulum and Golgi. This suggest that the first signalling events triggered by the toxin on cell surface could be involved in NCL secretion mechanism.

The type of interaction between NCL and Mt-II is also interesting. The region of NCL interacting with Mt-II is not its N-terminal negatively charge domain, as one would expect from the fact that Mt-II has a very high isoelectric point, but its central RNA binding domain and the C-terminal RGG domain. Moreover, NCL and Mt-II colocalize in large areas of the cell surface and Mt-II seems to form amyloid-like structures in contact with cells. This suggests that the NCL/Mt-II interaction is not one-to-one but a multi-molecular assembly. NCL, rich in disordered regions and low complexity domains, is a typical protein participating in multimolecular interactions and phase transition phenomena, that is, transitions from dispersed protein solutions to liquid-like phase-separated compartments or to solid protein aggregates^16,24^. Mt-II, like other sPLA_2_s, has a well-defined and compact 3D structure stabilized by seven disulphide bonds, so it is not expected to be inclined to form multimolecular interactions. However, we observed that Mt-II possesses exposed loops enriched in prion-like amino acids^28^, and several proteins involved in phase transition phenomena possess prion-like domains that contribute to the protein assembly^16,24^. Remarkably, NCL R/FGG domain, that we found involved in interaction with Mt-II, is similar to the glycine rich domains of the chaperones involved in yeast prion propagation^49^, moreover NCL is also a receptor of amyloid beta peptide 1–42^50^.

Other proteins can be involved in this multimolecular assembly, nucleophosmin for example, a known partner of NCL, that we identified among proteins co-precipitated by Mt-II (Table S4). Transmembrane proteins could be also involved, which may not have been detected in our fishing experiments since they are more difficult to isolate and identify by mass spectrometry. As NCL is not a membrane protein, it could communicates with the cell interior by means of transmembrane proteins, and, at this regard, both NCL and PLA2G2A were reported to interact with integrins and EGFR^38,51–53^.

Phospholipids are probably involved in the Mt-II multimolecular assembly too. In fact, sPLA_2_s were reported to form amyloid-like fibrils when they are exposed to phospholipid interfaces^27^ and Mt-II, even if catalytically inactive, is able to interact specifically with lipids^9^. sPLA_2_s share with antimicrobial and amyloid like peptides the ability to interact with and disrupt bacterial lipid membrane^54–56^. Moreover, antimicrobial peptides and PLA_2_ enzymes can form amyloid-type co-fibrils in presence of the PLA_2_ lipid hydrolysis products^57^, indicating that the PLA_2_ catalytic activity synergizes with the propensity to form amyloid-like fibrils. This can contribute to explain the synergy between catalytic and noncatalytic PLA_2_s, as observed for *B. asper* myotoxins I and II^58^.

Why should sPLA_2_s form this multimolecular assembly in cell surface? Code *et al*.^27^ suggested that amyloid-type formation of PLA_2_, in phospholipid bilayers, is functional to the control of enzyme action. We add that the function, on cell surface, could be also of triggering the endocytic process. Phase transition in cell surface could be useful to select and concentrate secreted protein factors with consequent activation of a signaling cascade, membrane movements and internalization mechanisms.

In conclusion, with this work, we demonstrated for the first time that *B. asper* Mt-II is internalized in cells and that its internalization and toxic activity depends on the interaction with the central RNA binding and the C-terminal domain of NCL. We think that this result, by focusing on the possible intracellular activity of *B. asper* Mt-II, will contribute to the understanding of the mechanism of action of this snake myotoxin.

## Material and Methods

### Isolation of Mt-II from *Bothrops asper* venom and modification with transglutaminase

Mt-II was isolated from the crude venom of *Bothrops asper*, a pool obtained from at least 30 specimens kept at the serpentarium of Instituto Clodomiro Picado, University of Costa Rica, as described in a previous work^58^. The isolated Mt-II was modified by reaction with transglutaminase from *S. mobaraensis* (Ajinomoto Co., Tokyo, Japan), as described in Spolaore *et al*.^20^, to obtain a protein mono-derivative with a fluorophore, for the imaging experiments, or with biotin for the fishing experiments. Briefly, toxin was dissolved at a concentration of 1 mg/ml in 0.1 M sodium phosphate buffer (pH 7), to this solution the reactive Z-Gln-Gly-CAD-DNS, Z-Gln-Gly-CAD-TAMRA or Z-Gln-Gly-CAD-Biotin (ZEDIRA GmbH, Darmstadt, Germany) (stock solution 34 mg/mL in DMSO) was added at a protein/ligand molar ratio of 1/20. TGase was added at an enzyme/substrate (E/S) ratio of 1/25 (w/w), and the reaction mixtures were incubated for 4 hours at 37 °C. Reactions were stopped by addition of iodoacetamide (100 μM final concentration) that inactivates TGase enzyme by carbamidomethylation of the cysteine 64 catalytic residue. The fraction of mono-labeled Mt-II was purified from the reaction mixture by RP-HPLC with a C18 column (150 × 4.6 mm, 5 µm particle size; Phenomenex). The obtained product was lyophilized with a Freeze Dryer Edwards E2-MS (Milano) and analyzed by a Q-Tof Micro mass spectrometer (Micromass, Manchester, UK).

### Cell cultures, cytotoxicity and internalization assays

The murine macrophage cell line RAW264.7 (ATCC TIB 71) and skeletal muscle C2C12 (CRL-1772) were obtained from the American Type Culture Collection and were maintained in Dulbecco’s modified Eagle’s medium (DMEM) supplemented with heat-inactivated fetal bovine serum (EuroClone) 10%, 100 µg/ml streptomycin, 100 U/ml penicillin. Primary cultures of skeletal muscle cells were prepared from newborn (1–2 days-old) mice (CD1) as reported by Massimino *et al*.^59^ and seeded in Ham’s F12 (Eurobio) supplemented with 10% (v/v) fetal calf serum, 2 mM glutamine, 100 U/ml penicillin, 100 µg/ml streptomycin (Eurobio). Newborn mice were taken from our facility. All aspects of animal care and experimentation were performed in compliance with European and Italian (D.L. 26/2014) laws concerning the care and use of laboratory animals. All experimental procedures and animal care protocols were approved by the Italian Ministry of Health, and by the Ethical Committee for animal care and use of the University of Padova (*OPBA, Organismo Preposto al Benessere degli Animali*).

For C2C12 and primary myoblasts differentiation, the growth medium was replaced with DMEM containing 2% (v/v) horse serum (Eurobio) and the cells were incubated for 5–6 days.

For the cytotoxicity assay macrophages and differentiated myotubes were grown in 96-well plates and then exposed to Mt-II (20 µg/ml), with or without the indicated competitor, dissolved in modified Krebs–Ringer buffer (mKRB) (140 mM NaCl, 2.8 mM KCl, 2 mM MgCl_2_, 1 mM CaCl_2_, 10 mM Hepes, and 11 mM glucose, pH 7.4) for the indicated time. Cellular necrosis of primary mouse myotubes was estimated by measuring the release of LDH using the commercial kit TOX7 (Sigma). RAW264.7 cell death was evaluated with the colorimetric MTT assay, CellTiter96 (Promega). All antagonists were checked for their effect on cell viability and used at non-toxic concentrations.

For the myotoxin internalization assay RAW264.7 cells or primary mouse myotubes grown in 96-well plates were incubated with Mt-II-TAMRA (15 µg/ml) in presence of different inhibitors for 10 minutes. The cells were then washed three times with ice-cold PBS to remove the non-internalized protein and lysed with 100 µL of lysis buffer (50 mm Tris, 0.8% Triton X-100, 0.2% SDS, pH 7.4). Cell-associated fluorescent protein was determined by measuring the fluorescence of the lysate using a TECAN Infinite M1000 microplate reader (Ex 547 nm and Em 573 nm). Basic absorption of Mt-II-TAMRA on cell surface was determined by treating cells at 4 °C with the same quantity of protein, to prevent the internalization process, and executing the same procedure. Cell-associated protein values were corrected subtracting the basic absorption to the cell surface, obtaining the quantity of internalized protein. All data were collected in triplicate and four independent experiments were performed.

For Mt-II polymerization status analysis on primary myotubes (4·10^4^ cells cells/well p96, 6 differentiation days), cells were treated with Mt-II (14 µg/ml) for 30 min, 4 °C. Then cells were washed with mKRB and treated (60 min, 4 °C) with different quantities of the cross-linker BS3 (Thermo Scientific), resuspended in Laemmli Sample Buffer and analyzed by western blot (Bolt™ 4–12% Bis-Tris Plus Gels, Invitrogen) with conjugated streptavidin-HRP (1:1000, Invitrogen).

HeLa cells were cultured in standard conditions in DMEM added with 10% FCS and 2 mM glutamine and antibiotics. RNA interference of NCL was achieved using NCL Trilencer-27 Human siRNA, using a non-targeting duplex siRNA as control, all purchased from Origene. Immediately before transfection 6000 cells were seeded in each well of 96-well plate in 100 μl of standard medium without antibiotics. A volume of 0.1 μl/well (20 μM solution in water) of each siRNA was added to 20 μl of Opti-MEM I reduced serum medium (Thermo Fisher Scientific), incubated 5 minutes at RT and subsequently 0.16 μl of RNAiMAX Transfection Reagent (Thermo Fisher Scientific) were added. The mix reaction was incubated for 15′, RT, and then added to cells. 48 h from transfection, cells were treated with 10 and 20 ug/ml of Mt-II dissolved in mKRB and after 45′ analyzed for cell vitality (MTT assay) and resuspended in Laemmli Sample Buffer to assess the quantity of NCL by western blot.

Oligodeoxynucleotides AS1411, 5′-d(GGTGGTGGTGGTTGTGGTGGTGGTGG) and CRO, 5′-d(CCTCCTCCTCCTTCTCCTCCTCCTCC) were purchased from Invitrogen, the F3 peptide (AKVKDEPQRRSARLSAKPAPPKPEPKPKKAPAKK) was synthesized by Fmoc (9-fluorenyl methyloxy-carbonyl)-solid phase peptide synthesis.

### Fishing experiments and cross-linking on magnetic beads

Biotinylated Mt-II (Mt-II-B, 2 µg) was combined with 5 µl of Pierce Streptavidin Magnetic Beads (Thermofisher), pre-washed according to the manufacturer’s instruction. Mt-II-B and beads were incubated for 1 hour in a 2 ml low binding tube (Eppendorf) in Thermomixer (Eppendorf) at 25 °C, 500 rpm and then excess of Mt-II-B was washed with PBS. After each step of incubation or washing, beads were collected with a magnetic stand. Following steps of cell lysis were performed on ice, centrifugation was performed at 4 °C in a fixed angle rotor, incubations were performed in Thermomixer at 4 °C, 500 rpm, equilibration and elution at 25 °C, 500 rpm. RAW264.7 cells, seeded the day before in p6 (5 · 10^5^/well), were washed with mKRB and lysed in 200 µl/well of breaking solution (BS) (50 mM TRIS, 25% (w/w) sucrose, 5% (v/v) glycerol, 5 mM MgCl_2_, 2,8 mM KCl, 2 mM CaCl_2_ and Roche protease inhibitors) with 10 passages through a 25 G needle. The obtained suspension was briefly vortexed and left on ice for 30 minutes and then centrifuged for 3 minutes, 720 × g, to eliminate unbroken cells and most of the nuclei. The supernatant was collected and incubated overnight with 5 µl of streptavidin magnetic beads combined with Mt-II-B, or without Mt-II-B for background control. Then the beads were washed three times for 3 minutes each, in Thermomixer, with 200 µl of BS and three times with 200 µl of IP Buffer (Pierce, 25 mM Tris-HCl pH 7.4, 150 mM NaCl, 1 mM EDTA, 1% NP-40 and 5% glycerol). The beads were finally equilibrated with three passages in 200 µl of 50 mM NH_4_HCO_3_ (pH 8) and the isolated proteins were eluted with 20 µl of 5% sodium deoxycholate (SDC) in 50 mM NH_4_HCO_3_. In the third experiment the elution step was preceded by crosslinking with 100 µl of 0.25 mM 3,3′-dithiobis(sulfosuccinimidyl propionate) (DTSSP, Thermo Scientific), in 50 mM NH_4_HCO_3_, at 25 °C for 30 min. Reaction was stopped with 10 µl of 1 M TRIS pH 7.5 and beads were washed three times with 5% SDC in NH_4_HCO_3_ (at 25 °C) to remove non-crosslinked proteins. Finally, elution was performed with 50 mM DTT in 5% SDC, 50 mM NH_4_HCO_3_. Eluted proteins were analyzed by electrophoresis in 4–12% Bis-Tris Protein Gels (ThermoFisher) stained with SimplyBlue SafeStain (ThermoFisher).

Membrane protein extracts were obtained with a Membrane Protein Extraction Kit (BioVision, Milpitas, CA) by following the manufacturer’s instructions, starting from 5 · 10^8^ RAW264.7 cells, 1.5 · 10^8^ C2C12 myotubes or 4.5 g of mouse posterior limb muscles (from 1 day-old mice). Membranes were resuspended in 1.2 ml of BS and fishing was executed as reported in the previous paragraph, combining 200 µl of membrane suspension with 2 µg of Mt-II-B / 5 µl of streptavidin beads. Eluted proteins were analyzed by western blot probed with anti-NCL rabbit polyclonal antibody C23 H-250 (Santa Cruz), diluted 1:200. BlueStar pLUS Prestained Marker (Nippon Genetics Europe GmbH) was used as MW standard. IRDYE 800cw, Goat anti-Rabbit (LI-COR)(1:10^4^) and IRDYE 680cw, Goat anti-Mouse were used as secondary antibodies. Pictures were acquired with Odyssey CLx (LI-COR) and analyzed with Image Studio Software 4.0 (LI-COR). The kit efficiency was checked by western blot analysis of RAW264.7 subcellular fractions (see Supplementary Fig. S5).

### Mass spectrometry analysis

Samples isolated from the fishing experiments performed as described above were loaded in a 4–12% Bis-Tris Protein Gels (ThermoFisher). The electrophoretic separation was stopped after about 10 min, as soon as the protein extracts completely entered the running gel. This preliminary step allows the removal of salts and any other possible interfering compounds from the sample. Gel bands were then excised, cut in smaller pieces, washed several times with 200 µL of 50 mM NH_4_HCO_3_ (pH = 8) and dried under vacuum after a short wash with 200 µL of acetonitrile (ACN). Cysteines were reduced with 10 mM dithiothreitol (in 50 mM NH_4_HCO_3_) for 1 h at 56 °C, and alkylated with 55 mM iodoacetamide (in 50 mM NH_4_HCO_3_) for 45 minutes at room temperature (RT) in the dark. Gel pieces were then washed with alternate steps of 50 mM NH_4_HCO_3_ and ACN, and finally dried under vacuum. Proteins were in-gel digested with sequencing grade modified trypsin (Promega, Madison, WI, USA) at 37 °C overnight (12.5 ng/μL trypsin in 50 mM NH_4_HCO_3_). Peptides were extracted with three steps of 50% ACN/0.1% formic acid and the samples containing the peptide mixtures were dried under vacuum and stored at −20 °C until the LC-MS/MS analysis was performed.

LC-MS/MS analysis was conducted on an LTQ-Orbitrap XL mass spectrometer (ThermoFisher Scientific, Rockford, IL, USA), coupled with a nano-HPLC Ultimate 3000 (Dionex-ThermoFisher Scientific). Samples were loaded onto a pico-frit column (75 µm I.D., 15 µm tip, 11 cm length, New Objective) packed in house with C18 material (Aeris peptide 3.6 µm XB-C18, Phenomenex) and separated using a 45-minutes linear gradient of ACN/0.1% formic acid (from 3–40% ACN), at a flow rate of 250 nL/min. To avoid any possible carry-over effect on the chromatographic column, after each sample an identical analysis was performed by injecting a blank. The analysis was performed in a data-dependent mode: a full scan at 60,000 resolution on the Orbitrap was followed by MS/MS fragmentation scans on the four most intense ions acquired with collision-induced dissociation (CID) fragmentation in the linear trap. Raw data files were analyzed with the software MaxQuant (Cox, J. and Mann, M. *Nat Biotechnol*, 2008, 26, pp 1367–72) against the mouse section of the Uniprot database (version 20141201), concatenated with a small database of the most common contaminant proteins found in proteomics experiments. Enzyme specificity was set to trypsin with up to 2 missed cleavages. The mass tolerance window was set to 20 ppm for parent mass and to 0.5 Da for fragment ions. Carbamidomethylation of cysteine residues was set as fixed modification and methionine oxidation as variable modification. Results were filtered to keep into account only proteins identified with a false discovery rate (FDR) less or equal to 0.01 and with at least four independent unique peptides sequenced with high confidence by MS/MS. The intensity parameter calculated by MaxQuant was used to compare the abundance of the proteins isolated from the fishing in the presence of Mt-II with those obtained from the control experiment.

### Live imaging, immunofluorescence and Congo red staining of Mt-II intoxicated cells

RAW264.7 cells (1 · 10^5^ cells/13 mm, 5 · 10^5^/24 mm) or primary myotubes (3 · 10^5^/13 mm, 1.5 · 10^6^/24 mm) were seeded onto 13 mm coverslips coated with collagen 0.1% in HCl 0.01 M (only for primary myotubes). For internalization experiments (Fig. 1) mouse primary myotubes were pre-treated for 20 minutes (37 °C, 5% CO_2_) with Hoechst 33342 1:1000 in culture medium. Cells were treated with solution of Mt-II-TAMRA (15 µg/ml) in mKRB for 30 min, 37 °C, 5% CO_2_, washed 3 times with mKRB and observed with a LEICA CTR6000 epifluorescence microscope.

For colocalization experiments with surface NCL, RAW264.7 cells and myotubes were incubated with anti-NCL rabbit polyclonal antibody C23 H-250 (Santa Cruz) diluted 1/50 in mKRB for 45 minutes at RT, then treated with 15 µg/ml Mt-II-TAMRA in mKRB for 15 min, at 4 °C. After extensive washings in PBS, cells were fixed with PFA (2% in PBS) for 20 minutes 4 °C and incubated for 1 h, RT with the secondary antibody Alexa Fluor 488-conjugated anti-rabbit IgG (1:500, Molecular Probes). For amyloid-like structures, after Mt-II treatment, 14 µg/ml, 10 min, 4 °C, cells were stained for 20 minutes at RT with Congo red (Sigma) 0.02 mg/ml in PBS and 0.01% NaOH. Cell nuclei were counter-stained with Hoechst 33342 (5 µg/ml, Sigma). Finally coverslips were mounted in 8% Mowiol 40–88 (Sigma) in glycerol and PBS (1:3 = v/v).

For intracellular colocalization experiments with NCL, RAW264.7 cells and myotubes were incubated with Mt-II-TAMRA 15 µg/ml in mKRB for 20 min, at 37 °C. After extensive washings in PBS, cells were fixed with PFA (2% in PBS) for 20 minutes 4 °C, permeabilized with 0.5% Triton in PBS, and incubated with anti-NCL rabbit polyclonal antibody C23 H-250 (Santa Cruz) diluted 1/50, overnight at 4 °C, and with the secondary antibody Alexa Fluor 488-conjugated anti-rabbit IgG (1:500, Molecular Probes), for 1 h, RT.

Images were acquired with inverted fluorescence microscope (Leica CTR6000) equipped with a computer-assisted charge-coupled camera (Orca Flash 4.0, Hamamatsu), or with a confocal microscopy system (Leica TCS-SP5). Amyloid-like structures were observed under cross-polarized light with a DMR microscope (Leica). Acquisition and analysis of digital images were done with the Leica LAS AS software. Confocal reported images are a single plane of a complete z-stack of the observed field, composed of 10–15 sections taken with a step-size of 0.15–0.20 µm, pinhole [m] 67.9 µm, pinhole [airy] 1.00.

### Data availability

All data is provided in supplementary data materials.

## Electronic supplementary material

Supplementary Information

Table S1

Table S2

Table S3

Table S4
