# Supplementary material for: Cell surface nucleolin interacts with and internalizes Bothrops asper Lys49 phospholipase A2 and mediates its toxic activity
Source: Sci Rep. 2018 Jul 13;8:10619. doi: 10.1038/s41598-018-28846-4 (PMC6045611; doi:10.1038/s41598-018-28846-4)

Cell surface nucleolin interacts with and internalizes *Bothrops asper* Lys49 phospholipase A_2 and mediates its toxic activity

Maria Lina Massimino, Morena Simonato, Barbara Spolaore, Cinzia Franchin, Giorgio Arrigoni, Oriano Marin, Laura Monturiol-Gross, Julián Fernández, Bruno Lomonte, and Fiorella Tonello

Supplementary figures

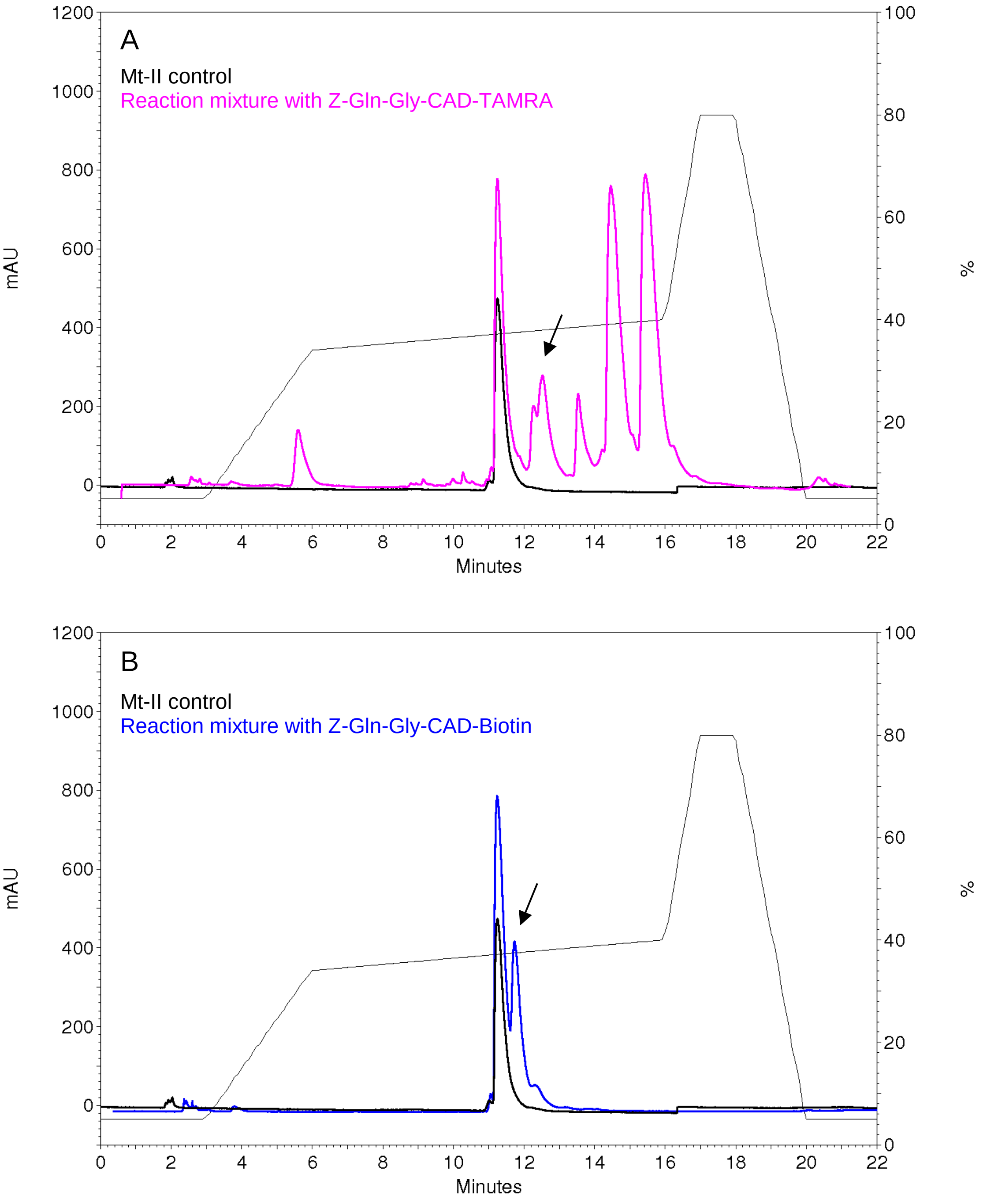

Figure S1. Reverse phase HPLC purification of Mt-II modified with TGase. The reaction mixture of Mt-II with Z-Gln-Gly-CAD-TAMRA (**A**) or Z-Gln-Gly-CAD-Biotin (**B**) in presence of TGase was loaded in a RP-HPLC C18 column and eluted with an acetonitrile gradient (black line in the chromatogram), monitoring the absorbance at 280 nm. The peaks were collected and characterized by ESI mass spectrometry (see Figure S2). The peaks indicated by the arrow correspond to the mono-modified proteins.

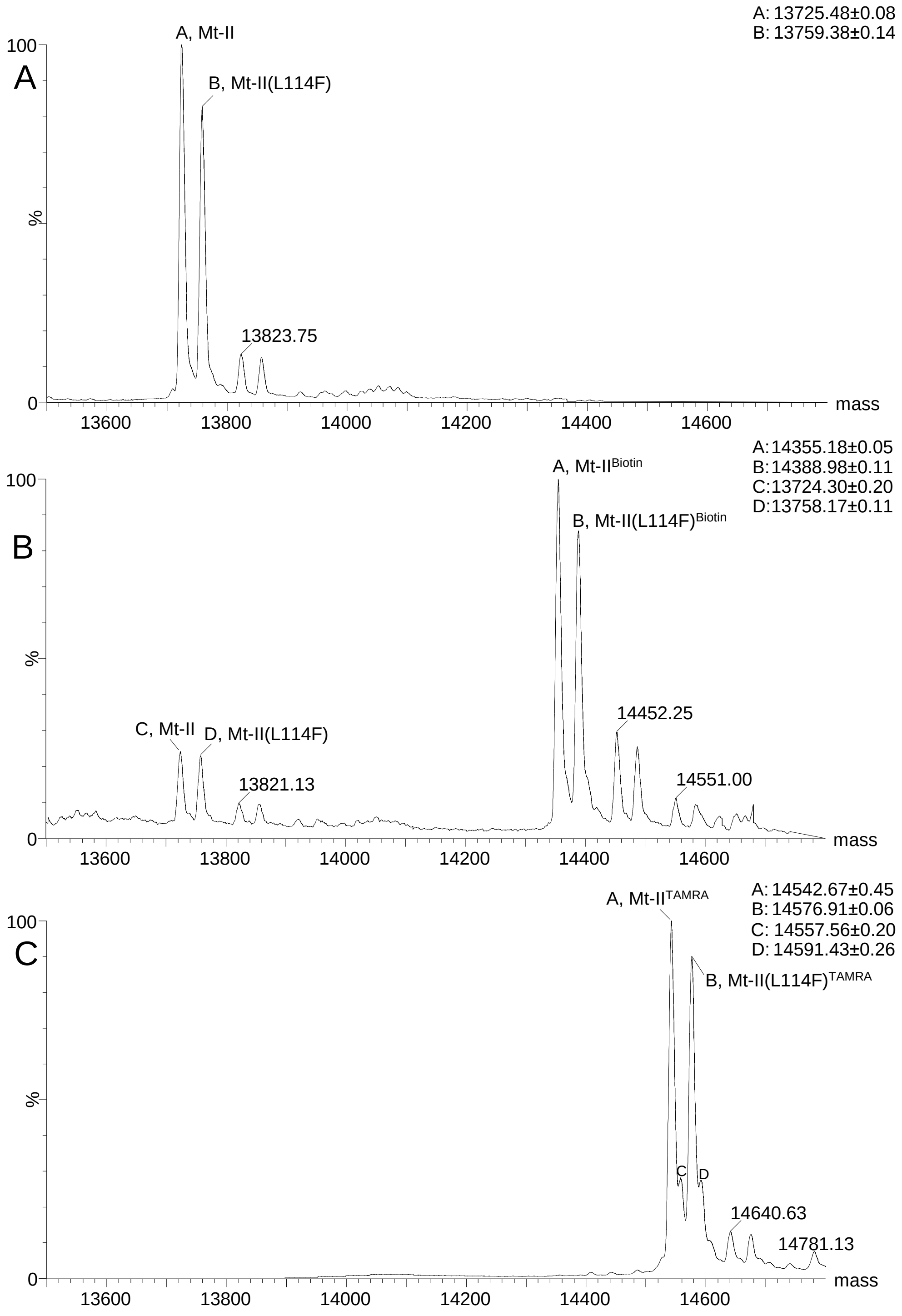

Figure S2. Deconvoluted ESI mass spectra of native Mt-II collected in the RP-HPLC analysis of the reaction mixture of Mt-II, Z-Gln-Gly-CAD-TAMRA and TGase after 0 h of incubation (A), and of the Mt-II derivatives collected in the RP-HPLC analyses of the reaction mixture with Z-Gln-Gly-CAD-Biotin (B) or Z-Gln-Gly-CAD-TAMRA (C) after 4 h of incubation. The derivatized Mt-II species are indicated by the superscript "Biotin" for the conjugation to one molecule of Z-Gln-Gly-CAD-Biotin and "TAMRA" for the modification with one Z-Gln-Gly-CAD-TAMRA. In spectrum C, species C and D likely correspond to Mt-II-TAMRA and Mt-II (L114F)-TAMRA oxidized forms, respectively.

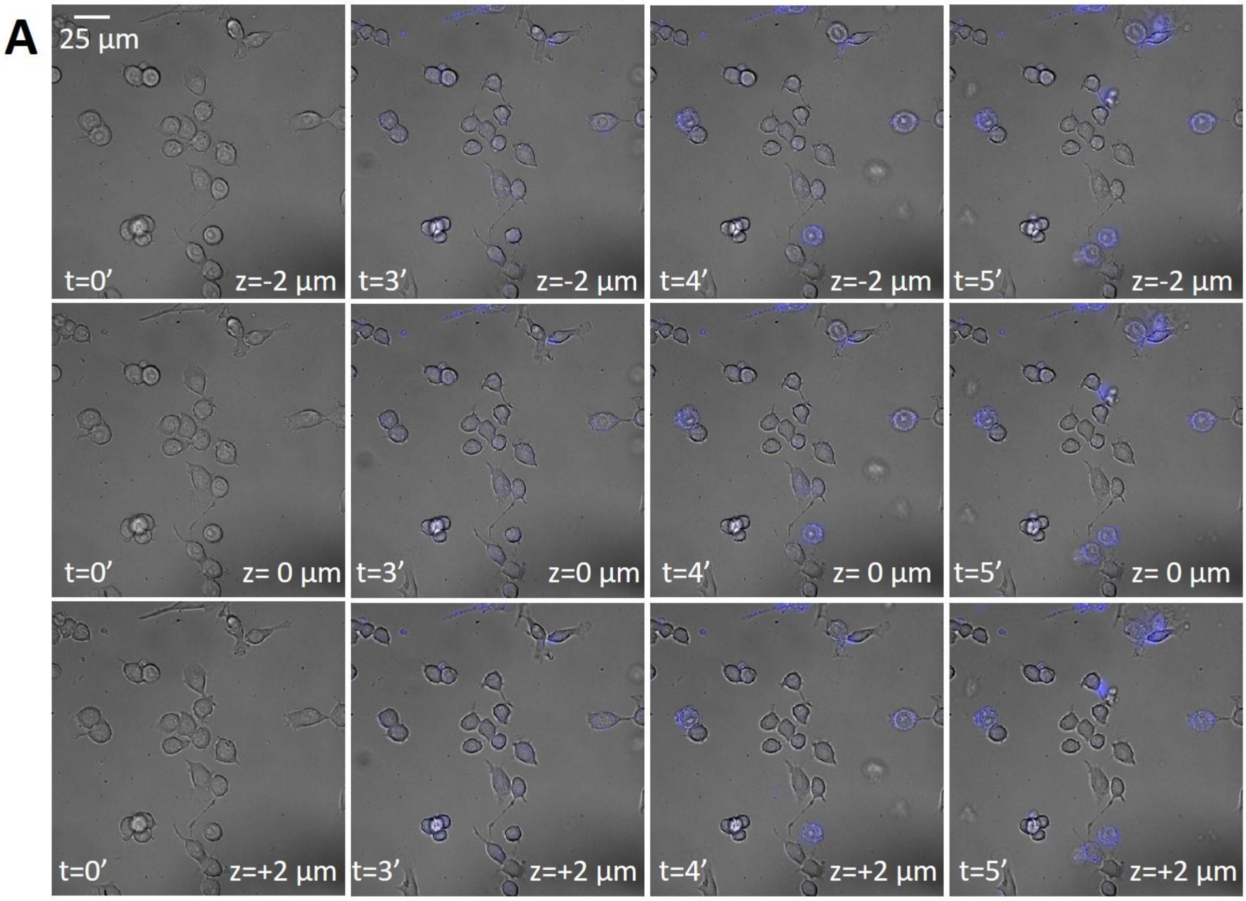
A
25 µm
t=0′
z=-2 µm
t=3′
z=-2 µm
t=4′
z=-2 µm
t=5′
z=-2 µm
t=0′
z= 0 µm
t=3′
z=0 µm
t=4′
z= 0 µm
t=5′
z= 0 µm
t=0′
z=+2 µm
t=3′
z=+2 µm
t=4′
z=+2 µm
t=5′
z=+2 µm

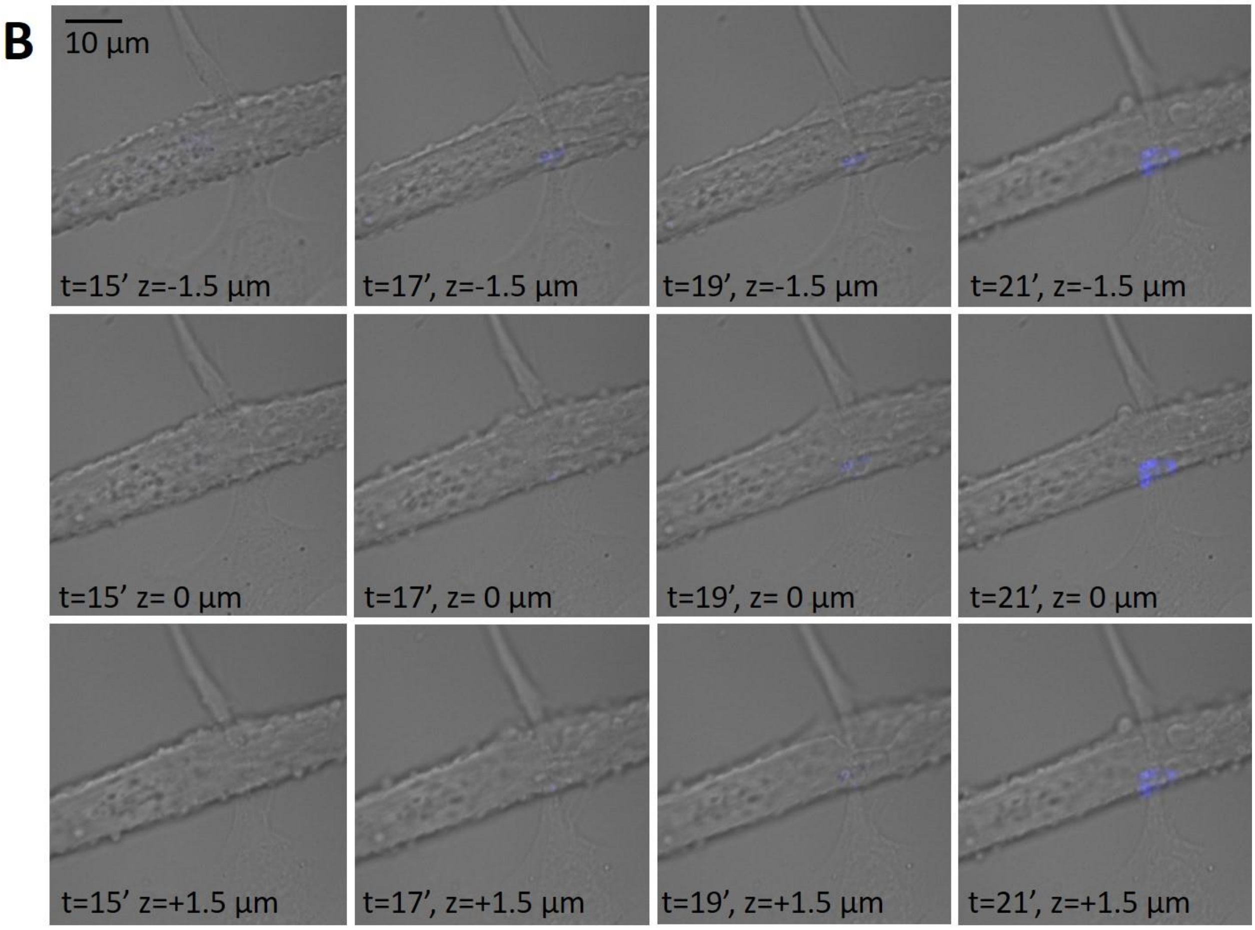

Figure S3. Time lapse images of RAW264.7 cells (A) and a mouse primary myotube (B) intoxicated with Mt-II conjugated with a DNS (blue) containing peptide. Mt-II was derivatized with Z-Gln-Gly-CAD-DNS (Zedira) as described for the production of the conjugates with Z-Gln-Gly-CAD-TAMRA and Z-Gln-Gly-CAD-Biotin. The measured masses of Mt-II modified with one Z-Gln- Gly-CAD-DNS (14361.99±0.34 Da for Mt-II and 14396.35±0.13 Da for the L114F isoform) were in agreement with the calculated mass (14362.8 Da and 14396.8 Da, respectively). Mt-II-DNS, 15 µg/ml, was added just after the t=0 point (not shown in the case of the myotube).

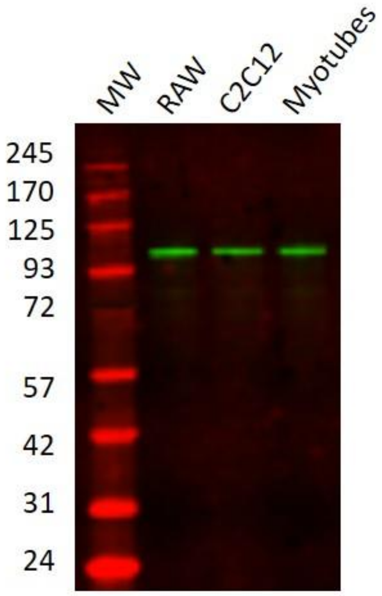

Figure S4. Mt-II pulls down NCL from membrane protein extracts. Western blot analysis, marked with anti-NCL, of proteins pulled down by Mt-II-B/streptavidin magnetic beads from RAW264.7 and mouse primary myotubes membrane protein extracts. MW standards: BlueStar pLUS Prestained Marker (Nippon Genetics Europe GmbH).

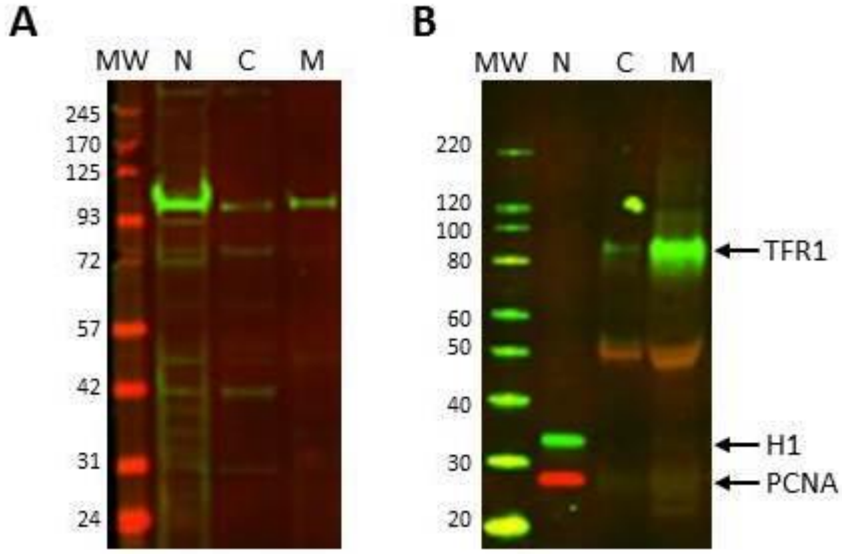

Figure S5. Analysis of RAW264.7 subcellular fractionation. Nuclear (N), cytosolic (C) and membrane (M) fractions were analyzed in western blot probed with (**A**) rabbit polyclonal (RP) anti-NCL C23 H-250 (Santa Cruz); (**B**) RP anti-transferrin receptor 1 (TFR1) (Abcam), RP anti-histone 1 (H1) (Abcam) and mouse monoclonal anti-proliferating cell nuclear antigen (PCNA)(BD Trasduction). MW standards: BlueStar pLUS Prestained Marker (Nippon Genetics Europe GmbH) (red) and MagicMark XP Western Protein Standard (Invitrogen) (green). As expected, NCL is present for > 90 % in the nucleus, and in lower quantities in cytosol and membrane fractions. H1 and PCNA, typical nuclear proteins, are not present in membrane fraction, marked with anti-TFR1 antibody.

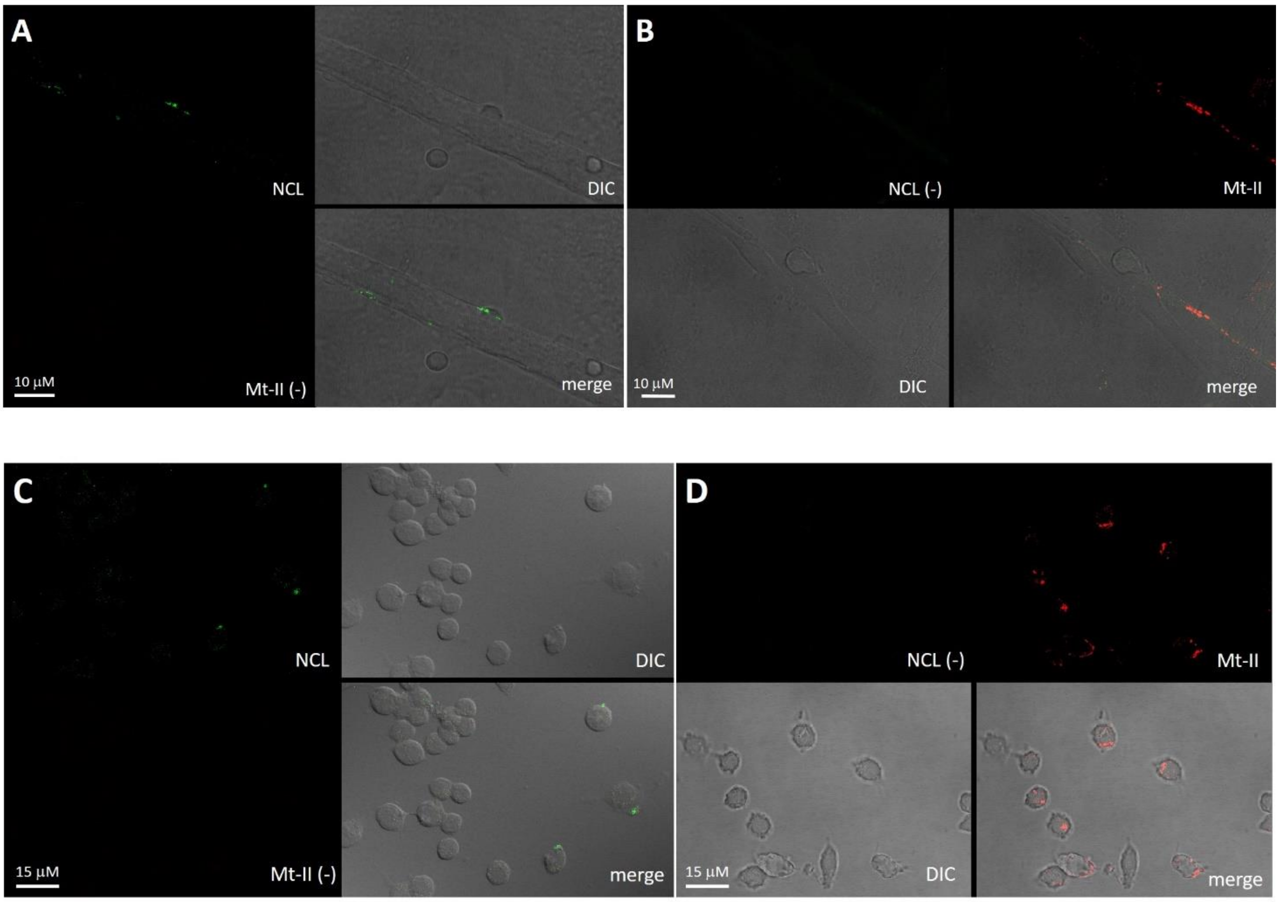

Figure S6. **Control images to figure Fig. 3**. Mouse primary myotubes (**A**, **B**) and RAW264.7 cells (**C**, **D**) were incubated with a rabbit anti-NCL (**A**, **C** green signal) or with Mt-II-TAMRA (**B**, **D** red signal), then fixed, treated with an anti-rabbit Ig secondary antibody Alexa Fluor 488-conjugated and visualized by confocal microscopy.

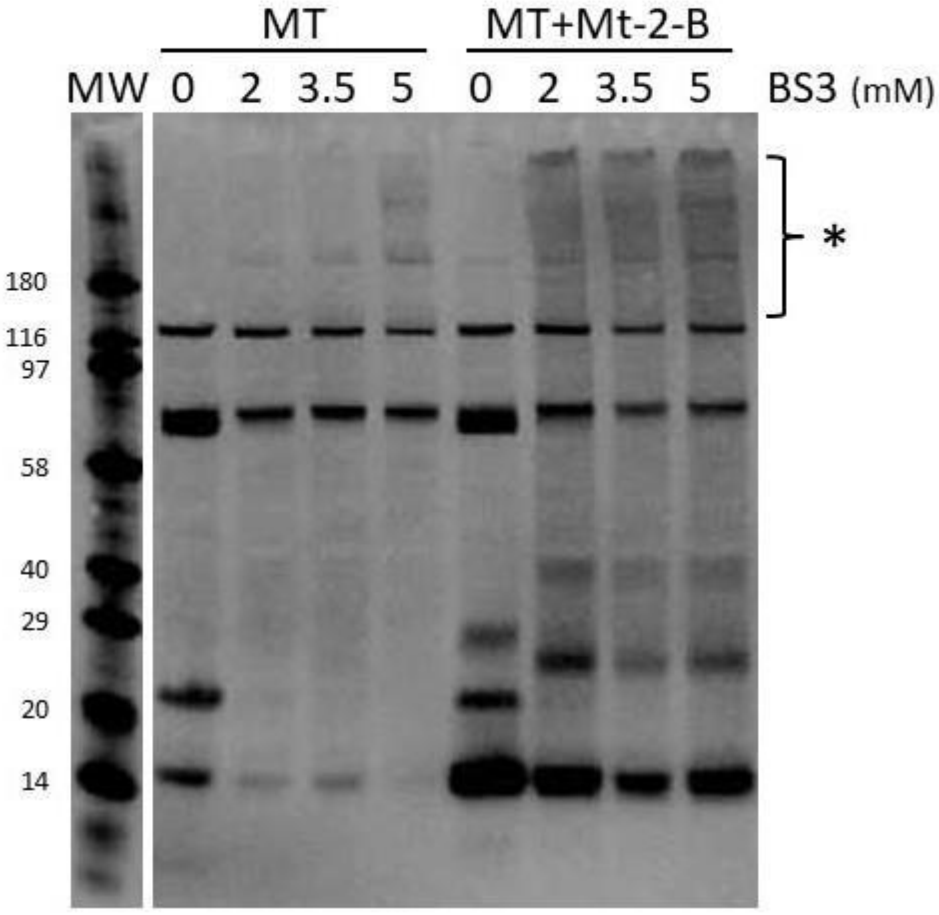

Figure S7. Oligomers formed by Mt-II on cell surface are not SDS-resistant. Primary mouse myotubes (MT) were incubated with control buffer or with biotinylated Mt-II (Mt-II-B, 15 µg/ml) for 30’, 4°C. Then cells were washed and treated (60’, 4°C) with different quantities of the crosslinker BS3, resuspended in Laemmli Sample Buffer and analysed in western blot with streptavidin-HRP (Invitrogen). A Biotinylated Molecular Weight Marker (Sigma) was used (MW). High molecular weight complexes, indicated by the brace (*), are present in samples containing Mt-II-B and treated with the crosslinking agent, but not in un-crosslinked samples.

A

```
B. asper Mt-II SLFELGKMILQETGKNPAKSYGAYGCNCGVLGRGKPKDATDRCCYVHKCCYKKL--TGCNPK 60
Human PLA2G2a  NLVNFHRMIKLTTGKEAALSYGFYGCHCGVGGRGSPKDATDRCCVTHDCCYKRLEKRGCGTK 62
Mouse PLA2G2a  NIAQFGEMIRLKTGKRAELSYAFYGCHCGLGGKGSPKDATDRCCVTHDCCYKSLEKSGCGTK 62
B. asper Mt-I  SLIEFAKMILEETKRLPFPYYTTYGCYCGWGGQGQPKDATDRCCFVHDCCYGKL--SNCKPK 60
               .: :: .**   * :     *  *** **  *:*.********* .*.***  *   .*  *

B. asper Mt-II KDRYSYSWKDKTIVCG-ENNSCLKELCECDKAVAICLRENLNTYNKKYRYYLKPLCKKADAC- 121
Human PLA2G2a  FLSYKFSNSGSRITCA-KQDSCRSQLCECDKAAATCFARNKTTYNKKYQYYSNKHCRGSTPRC 124
Mouse PLA2G2a  LLKYKYSHQGGQITCSANQNSCQKRLCQCDKAAAECFARNKKTYSLKYQFYPNMFCKGKKPKC 125
B. asper Mt-I  TDRYSYSRKSGVIICG-EGTPCEKQICECDKAAAVCFRENLRTYKKRYMAYPDLLCKKPAEKC 122
                  *.:* ..  * *. :   * ..:*:****.* *: .*  **. :*  * .  *:
```

B

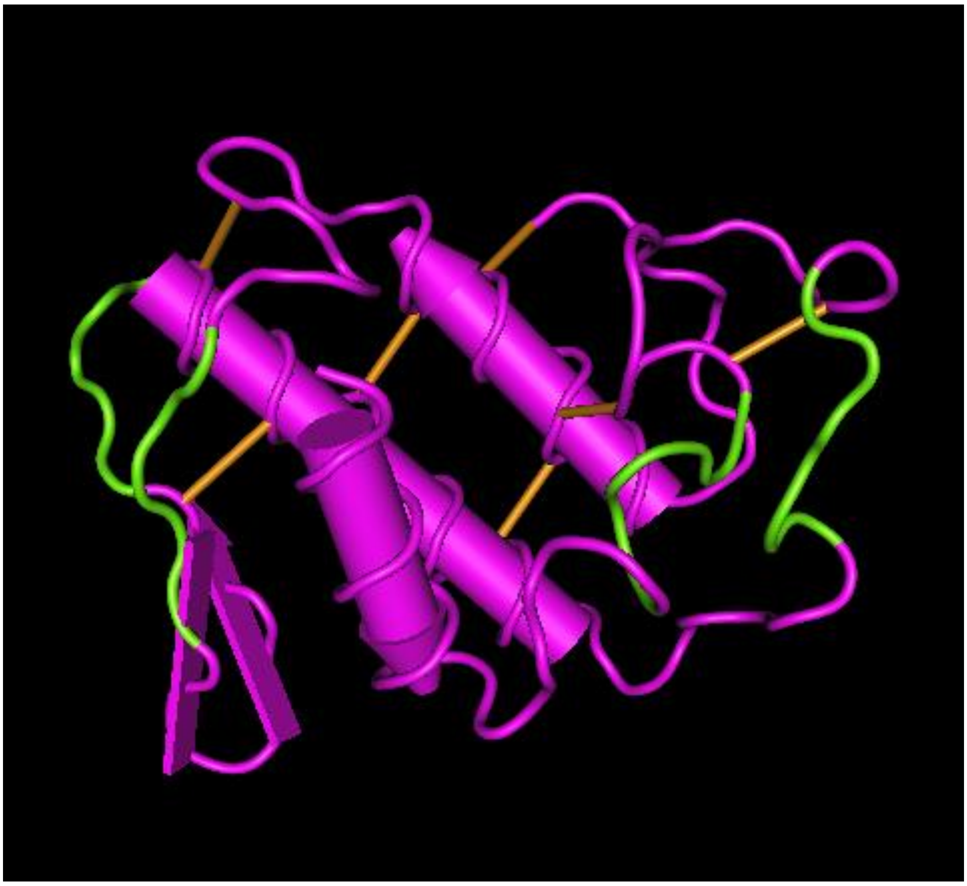

Figure S8. Presence of prion-like sequences on exposed loops of Mt-II and PLA2G2A. (**A**) Alignment of primary sequence of *B. asper* K49 myotoxin (Mt-II) and D49 myotoxin (Mt-I), human and mouse PLA2G2a. Secondary structure elements were underlined and traits rich in prion-like amino acids were evidenced in green. (**B**) Mt-II 3D model (PDB: 1CLP) where the traits rich in prion-like amino acids (see main text for definition) were colored in green. The picture was obtained with the Cn3D macromolecular structure viewing program.

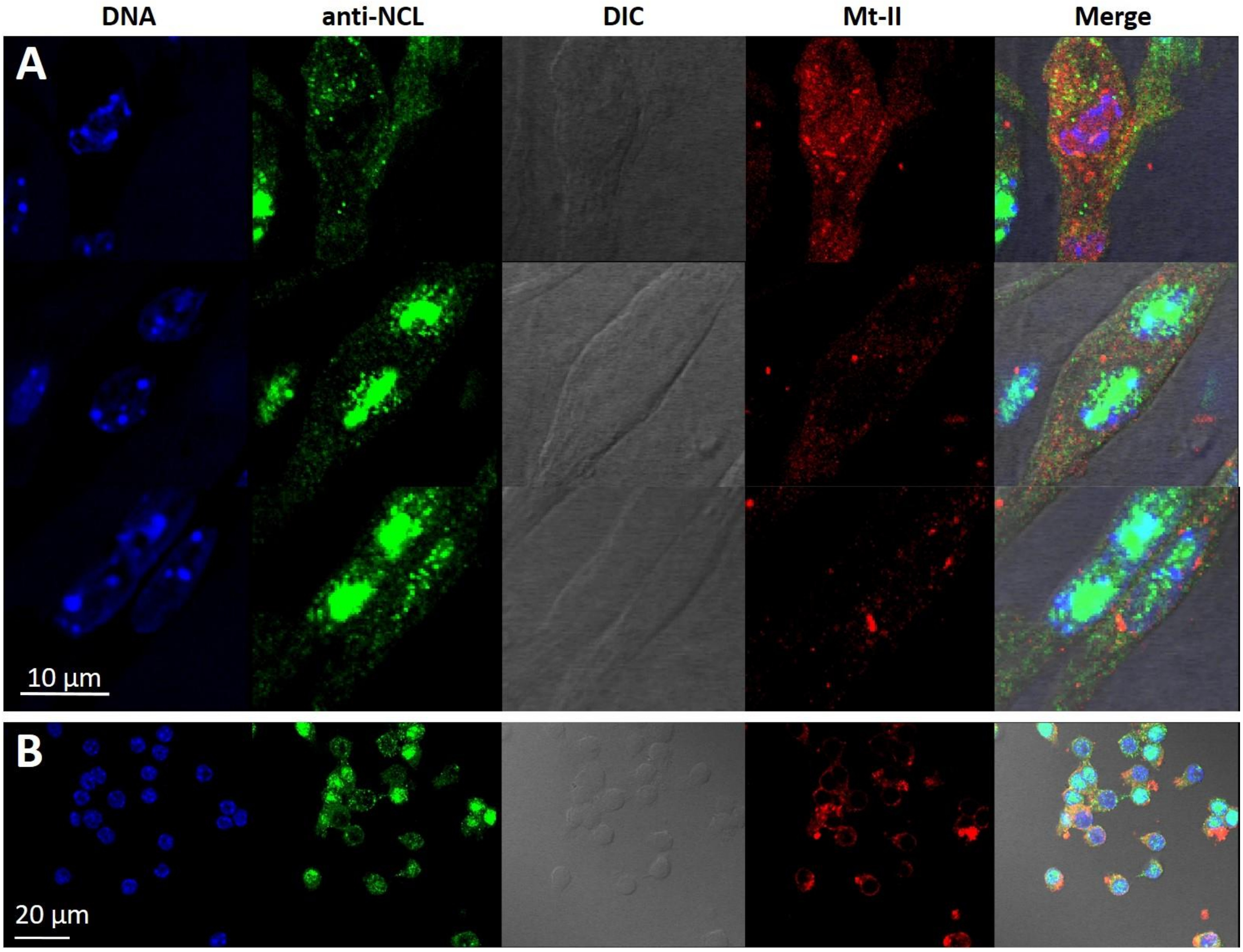

Fig. S9. **Intracellular colocalization of NCL and Mt-II in myotubes and macrophages.** Mouse primary myotubes (**A**) and RAW264.7 cells (**B**) were intoxicated with Mt-II-TAMRA (15 µg/ml) for 20', fixed, permeabilized with 0.5% Triton in PBS, and treated with a rabbit polyclonal anti-NCL and with an anti-rabbit Ig secondary antibody Alexa Fluor 488-conjugated and visualized by confocal microscopy. DNA were stained with the nuclear fluoro-probe Hoechst 33342.

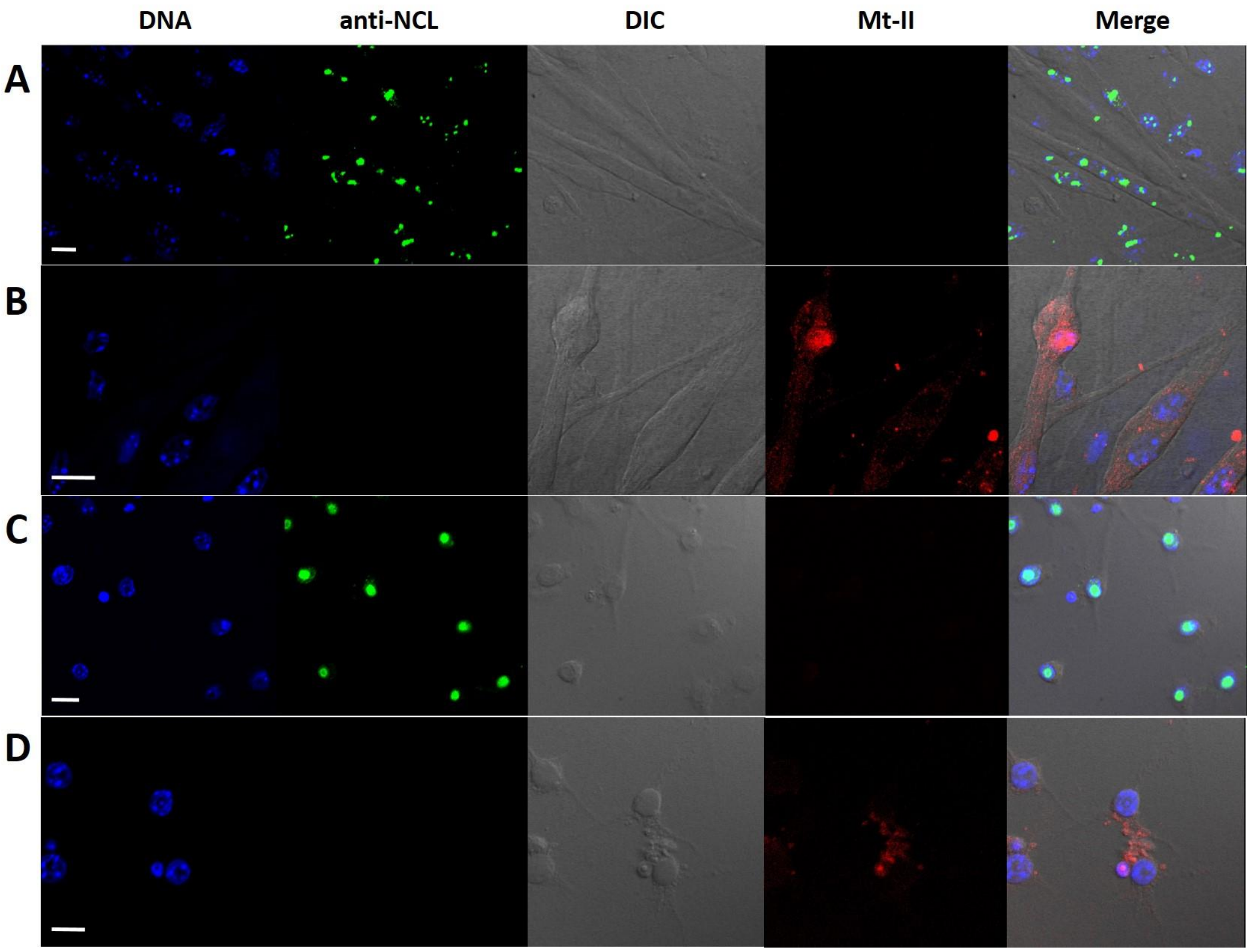

Figure S10. Control images to Fig. 5 and Fig. S9. Mouse primary myotubes (**A, B**) and RAW264.7 cells (**C, D**) were incubated with a rabbit anti-NCL (A, C green signal), or with Mt-II-TAMRA (B, D red signal), then fixed, treated with an anti-rabbit Ig secondary antibody Alexa Fluor 488-conjugated and visualized by confocal microscopy. DNA were stained with the nuclear fluoro-probe Hoechst 33342. Scale bars correspond to 10 µm.

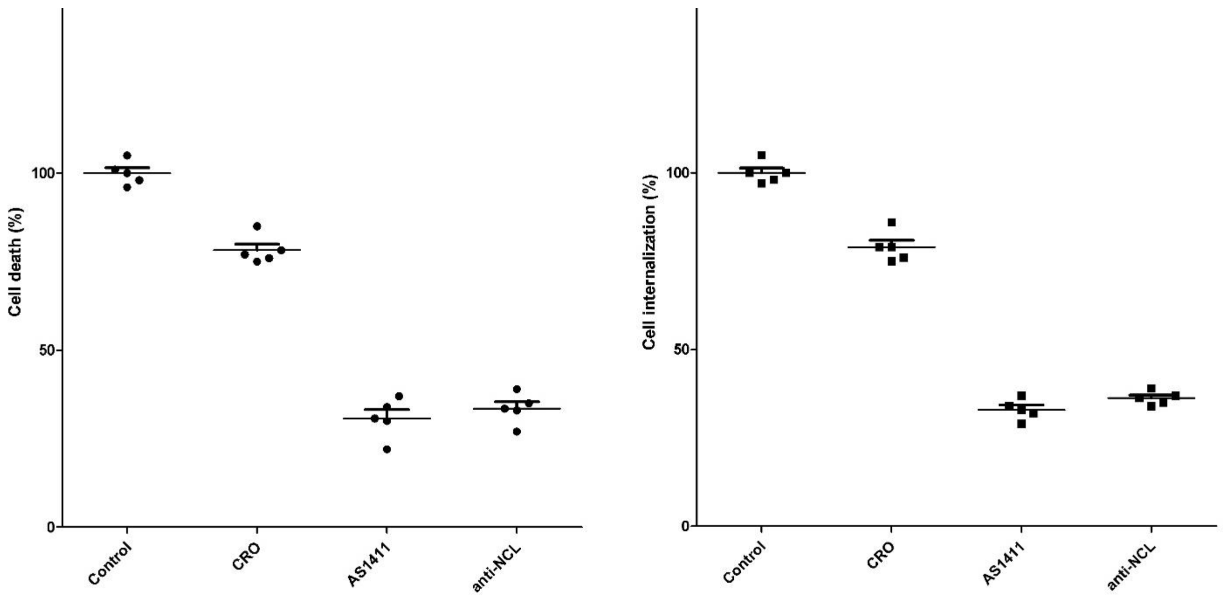

Figure S11. Inhibitory activity of NCL antagonists in cells intoxicated with Mt-II. RAW264.7 cells were treated with Mt-II (20 µg/ml) for 30’ at 37°C or with Mt-II-TAMRA (15 µg/ml) for 10’, 37°C and checked for their vitality (**A**) or for the internalization (**B**) of the fluorescent toxin. The experiments were repeated in presence of the NCL specific aptamer AS1411 (5 µM), a control aptamer (CRO, 5 µM) and an anti-NCL antibody (20 µg/ml), and the percentage of residual activity was calculated respect to control (100%).

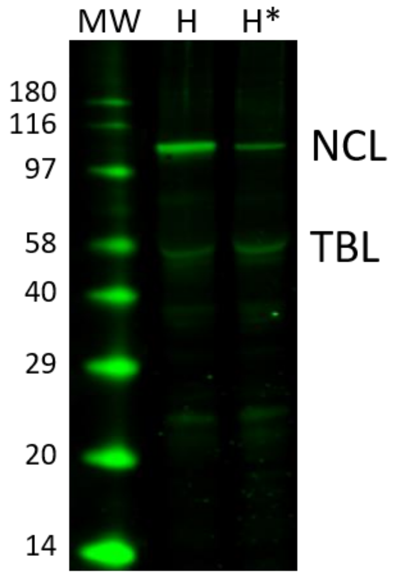

Figure S12. siRNA reduction of NCL expression in Hela cells. Western blot analysis of lysates of Hela cells treated with NCL Trilencer-27 Human siRNA (H*), or a non-targeting duplex siRNA control (H). The blot was marked with anti-NCL and with anti-tubulin as loading control.

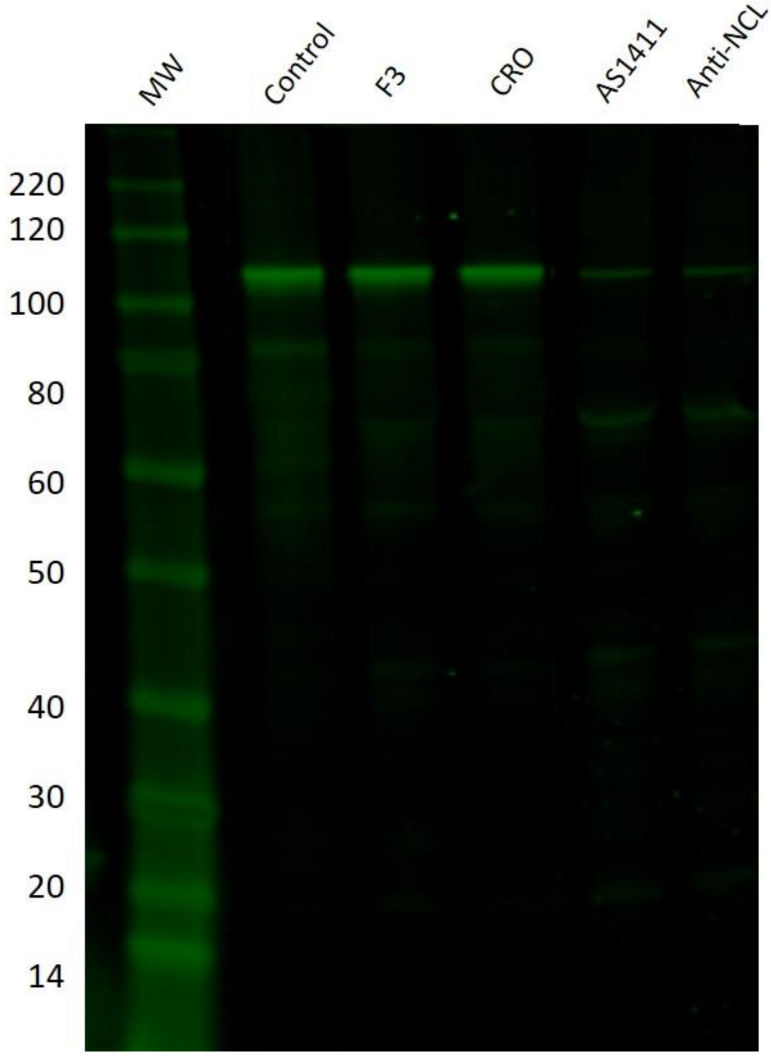

Fig. S13. **Mt-II interacts with NCL central and C-terminal regions**. Western blot analysis, marked with anti-NCL, of proteins pulled down by Mt-II-B/streptavidin magnetic beads from RAW264.7 membrane protein extracts, alone (control) or in presence of NCL binding molecules: a peptide (F3, 5 µM), an aptamer (AS1411, 5 µM), and an antibody (anti-NCL, 20 µg/ml). CRO is an aptamer used as control (5 µM). The experiment was executed as that reported in Figure 7A with the difference that in this case the cell membranes were re-suspended in 1.2 ml of 20 mM potassium HEPES, 50 mM potassium acetate, 15 mM Triton X100 pH 7,4 instead of the BS buffer (see the section ‘Fishing experiments and cross-linking on magnetic beads’ of the Matherials and Methods).

Original SDS-PAGE and western blot pictures:

Original picture of the SDS-PAGE presented in Fig. 2. Lane 2, 3, 6 and 7 are further control not described in the main article. The sample loaded in these lanes are the same loaded in lanes 4, 5, 8, 9 but with a control buffer as prey instead of the RAW264 cell extract.

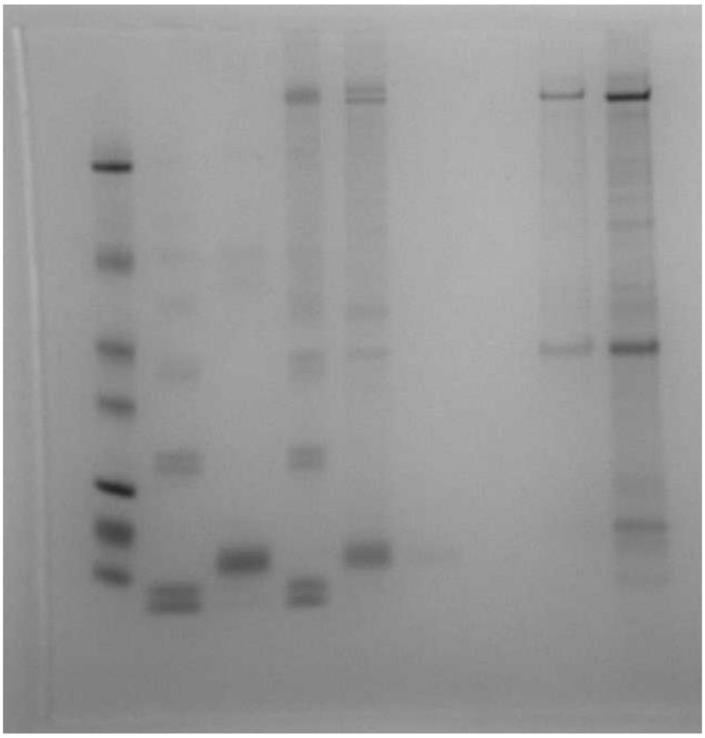

Original picture of the western blot presented in Fig. S6. Lane marked with an asterisk are further control not described in the article

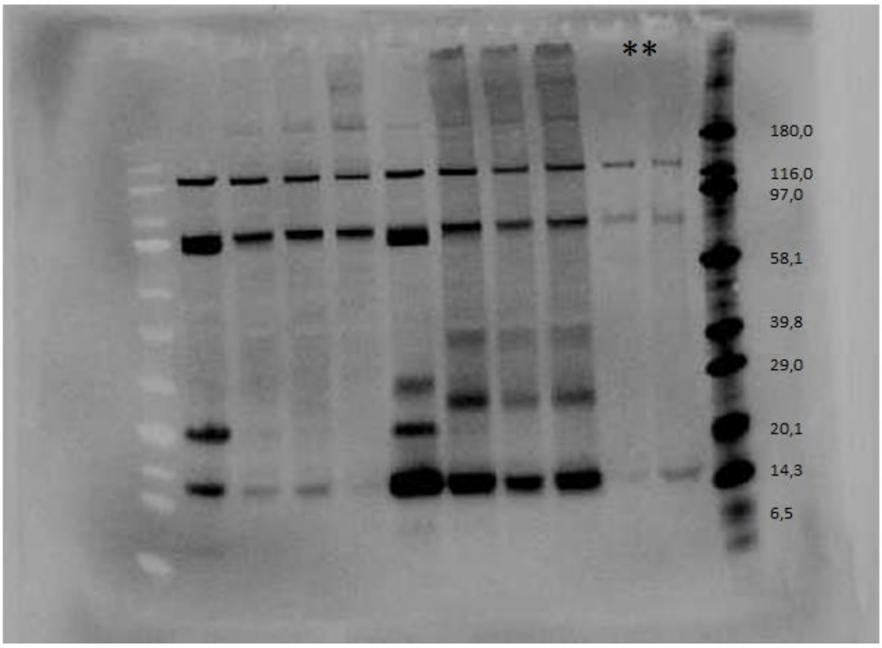

Full-length pictures of western blots presented in Fig. 7A, S3 and S4 A and B

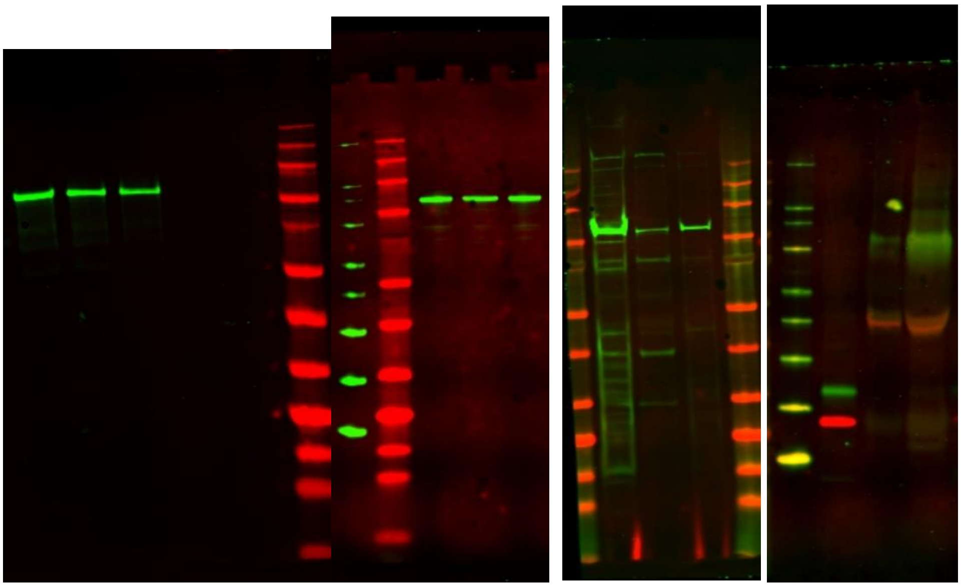

Supplement: Supplementary file 1 — Supplementary Information [file 41598_2018_28846_MOESM1_ESM.pdf]
